# Elevated Tolerance to Aneuploidy in Cancer Cells: Estimating the Fitness Effects of Chromosome Number Alterations by *In Silico* Modelling of Somatic Genome Evolution

**DOI:** 10.1371/journal.pone.0070445

**Published:** 2013-07-24

**Authors:** Anders Valind, Yuesheng Jin, David Gisselsson

**Affiliations:** Department of Clinical Genetics, Lund University and Skåne Regional and University Laboratories, Lund, Sweden; Virginia Tech, United States of America

## Abstract

An unbalanced chromosome number (aneuploidy) is present in most malignant tumours and has been attributed to mitotic mis-segregation of chromosomes. However, recent studies have shown a relatively high rate of chromosomal mis-segregation also in non-neoplastic human cells, while the frequency of aneuploid cells remains low throughout life in most normal tissues. This implies that newly formed aneuploid cells are subject to negative selection in healthy tissues and that attenuation of this selection could contribute to aneuploidy in cancer. To test this, we modelled cellular growth as discrete time branching processes, during which chromosome gains and losses were generated and their host cells subjected to selection pressures of various magnitudes. We then assessed experimentally the frequency of chromosomal mis-segregation as well as the prevalence of aneuploid cells in human non-neoplastic cells and in cancer cells. Integrating these data into our models allowed estimation of the fitness reduction resulting from a single chromosome copy number change to an average of ≈30% in normal cells. In comparison, cancer cells showed an average fitness reduction of only 6% (p = 0.0008), indicative of aneuploidy tolerance. Simulations based on the combined presence of chromosomal mis-segregation and aneuploidy tolerance reproduced distributions of chromosome aberrations in >400 cancer cases with higher fidelity than models based on chromosomal mis-segregation alone. Reverse engineering of aneuploid cancer cell development *in silico* predicted that aneuploidy intolerance is a stronger limiting factor for clonal expansion of aneuploid cells than chromosomal mis-segregation rate. In conclusion, our findings indicate that not only an elevated chromosomal mis-segregation rate, but also a generalised tolerance to novel chromosomal imbalances contribute to the genomic landscape of human tumours.

## Introduction

Over the last decade, a number of molecular mechanisms causing genomic alterations in cancer cells have been described. Structural aberrations of chromosomes, such as deletions, duplications and gene amplifications, are frequently caused by telomeric dysfunction or other triggers of DNA double strand breaks, followed by mitotic breakage-fusion-bridge cycles [1]–[3]. An unbalanced number of whole chromosomes (numerical aberrations; aneuploidy), on the other hand, is largely caused by mitotic spindle defects such as merotelic chromosome attachments [4], [5] or spindle multipolarity combined with cytokinetic failure [6]. Recently it has also been shown that chromosomes that mis-segregate can be damaged during cytokinesis, leading to DNA double strand breaks and unbalanced translocations in the daughter cells, thus implying an overlap between the routes leading to numerical and structural aberrations [7]. A prerequisite for the establishment of complex structural chromosome aberrations is tolerance to DNA double strand breaks, most particularly inactivation of the p53-dependent response [1], [8]. Overall, a tolerance to DNA breaks appears to be a very common feature in tumour cells when compared to non-neoplastic cells, allowing the mechanisms giving rise to genomic alterations to become established in tumours and enhancing the probability for tumorigenic mutations to occur [9].

A remaining question is whether cancer cells also react to novel changes in chromosome number, such as monosomies and trisomies, in a manner that distinguishes them from normal cells. If so, this factor could be just as important as mitotic spindle defects for the generation of aneuploidy in cancer. Some circumstantial evidence has been presented for an increased tolerance to novel chromosome aberrations in cancer cells. Non-neoplastic human cells have been shown to exhibit a significant rate of chromosome segregation errors at mitosis [6]. Because the prevalence of aneuploid cells nevertheless remains low in most somatic cells over the human lifespan, this indicates the presence of an endogenous negative selection pressure against (reduced fitness of) aneuploid cells in human tissues, as has been found in several model organisms [10], [11]. This negative selection may theoretically be reduced in cancer to allow aneuploidy on a broad scale. That eukaryotic cells may indeed develop such a tolerance against aneuploidy has recently been reported for a yeast model system, where aneuploidy-tolerating mutations were found to affect most prominently ubiquitin-proteosomal degradation pathways [12]. Taken together, these data beg the questions (1) whether a generalised tolerance to aneuploidy is present in human cancer cells, (2) what magnitude would such a tolerance have in cancer cells compared to normal cells, and (3) how would selective forces acting on aneuploidy affect the genomic landscape of tumours?

To our knowledge, few if any experiments have addressed these issues using primary human cells with comparisons to human cancer cells. One reason for this is that it is difficult to concurrently quantify chromosomal instability, aneuploidy and cellular proliferation parameters in growing human cells. An alternative approach is to carefully assess the rates of chromosomal mis-segregation as well as the exact prevalence of aneuploid cells in both cancer and normal cell populations, followed by incorporation of these data into a robust computational model of evolution on the cellular level. Using such a computational approach, we have investigated whether cancer cells have a higher tolerance to novel whole-chromosome alterations compared to non-neoplastic cells and to what extent such a reduced tolerance could explain the global pattern of chromosome alterations in neoplasia.

## Results

### Constructing Algorithms for Estimating the Impact of Aneuploidy on Proliferative Cell Survival

**Figure 1:**
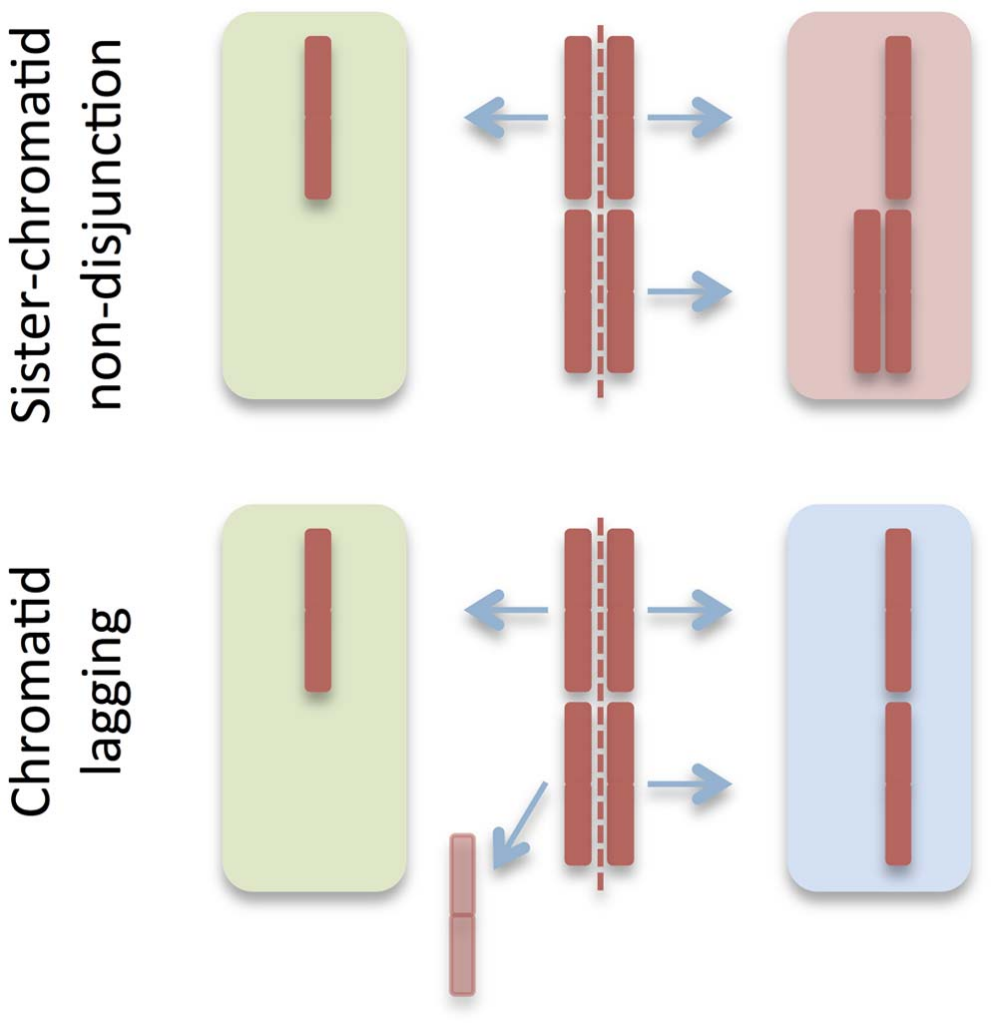
Major mechanisms of chromosome missegregation. Based on previous experimental data [6] mis-segregation was simulated as resulting from sister-chromatid non-disjunction resulting in one monosomic and one trisomic daughter cell (upper panel) and chromatid lagging resulting in monosomy in one daughter nucleus (lower panel). Each of these events was approximately equal in frequency, in all resulting in an average of 75% aneuploid daughter cells (red and green background) per mis-segregation event.

**Figure 2:**
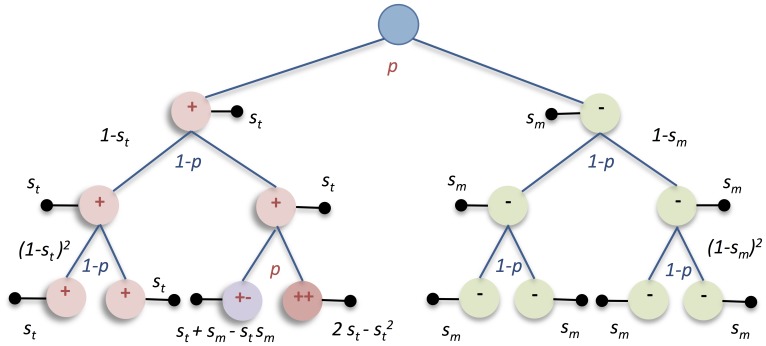
Example of the discrete time branching process in Chiron. Aneuploidy is generated at a specific rate (*p*) of mitotic mis-segregation, where trisomic (red circles) and monosomic cells (green circles) have certain probabilites (*s*_t_ and *s*_m_ respectively) of permanent proliferative arrest/death (black horizontal bars) at each mitotic cycle. Two or more aneusomies will result in increased probabilities of arrest/death as exemplified by cells labelled++and +−.

The main goal of the current study was to estimate the consequences of aneuploidy on long-term cellular survival in a human system, including both normal and cancerous cells. To achieve this, we constructed an algorithm that simulated the growth of a multi-cellular population from a single cell as a discrete time branching process of consecutive mitoses, each with a constant likelihood of chromosome segregation error (Figures 1 and 2). The main algorithm was implemented as the software Chiron (Figure S1; Protocol S1; Software S1 and S2), where the following parameters could be set arbitrarily: (1) the frequency of whole chromosome segregation errors per chromosome per mitosis; (2) the impact on proliferative survival of any autosomal whole chromosome aberration (monosomy, trisomy, tetrasomy etc.), except for nullisomy (0 copies of an autosome). Complete autosomal nullisomy was invariably considered lethal, because this type of aberration is observed very rarely in live human cells. The negative consequences of aneuploidy were specified by the selection parameter 0 ≤ *s* ≤ 1, in relation to the average proliferative survival of normal diploid cells. If *s = *0 for a specific chromosome aberration, then the proliferative survival of a cell carrying this aberration (*i.e.* being aneusomic) would be equal to that of the surrounding diploid cells, simulated as an equal probability of undergoing another mitosis. If *s = *1, implying maximum negative selection, the aneuploid cell would be excluded from all future mitotic generations. Values of *s* below 1 but larger than 0 imply a relative reduction of proliferative survival. For example, *s* = 0.4 equals a 40% probability of permanently exiting the mitotic cycle before the next cell division, given that the probability of another mitosis for diploid cells is 100%, *i.e.* a 60% reduction of proliferative survival.

**Figure 3:**
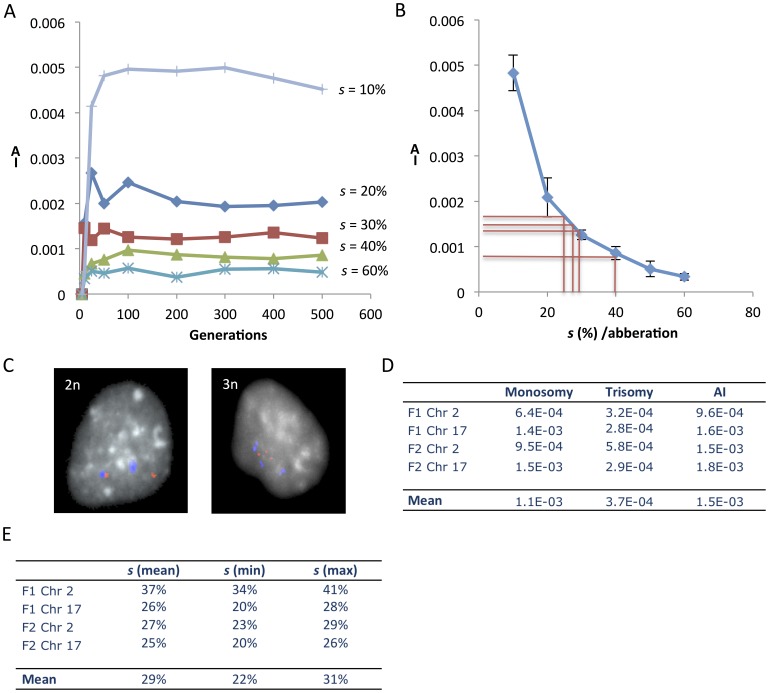
Estimation of negative selection acting on aneuploid cells in normal human fibroblasts. (A) Chiron-based simulation (20 parallel runs) of a growing cell population with a mis-segregation rate of 4×10^−4^ where different degrees of selection (*s*) against aneuploid cells are imposed. The resulting mean prevalence values of cells aneusomic (non-disomic) for a certain chromosome are given as aneusomy indices (AI) on the y-axis. AI is reduced with higher *s* values and reaches a dynamic equilibrium around 25 generations even at selection values as low as 10%. (B) Relationship of mean AI for a certain chromosome and negative selection acting on cells with aneusomy for this chromosome. Red lines correspond to estimations of selection made from experimental data for chromosomes 1 and 17 in F1 and F2. Error bars correspond to standard deviations in 20 simulations. (C) Fluorescence *in situ* hybridisation (FISH) was used to estimate AI in human normal fibroblasts, exemplified by F1 cells disomic (2n) and trisomic (3n) for chromosome 17 (red, q arm probe signal; blue, centromeric signal). (D) Prevalence of monosomic and trisomic cells as well as overall AI estimated by FISH in the two fibroblast lines F1 and F2. (E) Chiron-based estimates of the degree of negative selection acting on cells aneusomic for chromosomes 2 and 17 in F1 and F2, deduced by comparing FISH-data (AI) with modelling of AI as a function of *s* (Figure 3B). The max and min values correspond to ±2 standard deviations.

To monitor the inherent properties of this model, we first performed simulations with the rate of mitotic segregation errors fixed to the order of magnitude found in non-neoplastic human cells. In a previous study [6], we reported a median mis-segregation rate of 4×10^−4^/chromosome/mitosis (range 3.3−4.1×10^−4^) in primary human low-passage fibroblast cells, with a 2∶1 relationship between symmetrical nondisjunction of chromatids and chromatid lagging (Figure 1). This mis-segregation rate was similar in magnitude to previously reported rates for non-neoplastic immortalized human cell lines, derived from foetal or postnatal fibroblasts and/or epithelia [4], [13], [14]. Using a constant mis-segregation rate of 4×10^−4^/chromosome/mitosis, the prevalence of aneusomic cells was studied as a function of different degrees of negative selection against aneusomy for any chromosome in a cell population expanded for 500 generations. As expected, the prevalence of cells with aneusomy was inversely proportional to the degree of negative selection. After approximately 25 generations simulations with *s* ≥ 10% reached a plateau, indicating that a state of dynamic equilibrium had been reached between the generation of aneusomic cells and their elimination by negative selection (Figure 3A). Because 25 post-zygotic generations will generate a maximum of 3.36×10^7^ cells (not counting cell death and formation of extra-embryonic tissues), in turn corresponding to a solid biomass in the range of only 18–140 µL (assuming a cell diameter range of 10–100 µm), the dynamic equilibrium can be assumed to have presented well before birth for selection levels ≥ 10%. Hence, for a somatic cell lineage with a fairly constant level of chromosome mis-segregation, its frequency of aneusomic cells can be used to estimate the degree of aneusomy-dependent negative selection for a certain chromosome unless this selection value is very small (Figure 3B).

### Estimating Aneuploidy Levels and Aneuploidy Tolerance in Normal Cells

To estimate the degree of reduced fitness resulting from autosomal aneusomy in normal post-natal human cells by probabilistic modelling, we then determined the prevalence of aneusomic cells in fibroblast populations from two prepubescent individuals (F1 and F2) where mis-segregation rates had previously been estimated [6]. Earlier studies of aneuploidy prevalence in human cells from healthy tissues have shown vastly different results, even within the same cell type [15]–[19]. These variations could to some extent be attributed to the relatively high background of false positives for the type of analysis used (fluorescence in situ hybridisation, FISH), when employing a single probe to evaluate a chromosome for which aneusomy is present only in a small number of cells. To separate the presumably low level of aneusomy in normal cells from background caused by unspecific hybridisation, we used two differentially labelled FISH probes for each chromosome investigated in F1 and F2, targeting the centromere and a chromosome arm, respectively. This approach allowed not only identification of false positives due to cross-hybridisation of a single probe, but also subtraction of background levels resulting from erroneous hybridisation of two differently labelled probes (double false positives; Figure S2). To control for potential differences in impact between aneusomies for chromosomes with different size and gene content [15], we chose chromosomes 2 (≈243 Mb; 10,496 transcript alignments) and 17 (≈81 Mb; 6,330 transcript alignments) as index chromosomes for evaluation by interphase FISH (Figure 2C).

Estimation of the prevalence of cells aneusomic for chromosomes 2 and 17 in F1 and F2 by this approach resulted in an average prevalence of aneusomic cells of 1.5×10^−3^ per chromosome pair after background subtraction (Figure 2D). This corresponded to a prevalence of aneuploid cells of about 3%, when extrapolated to all chromosomes ( = 23 chromosome pairs). There was no significant difference in aneusomy prevalence between the fibroblast populations or between the two index chromosomes. The aneusomies observed were restricted to monosomies and trisomies, where the former were at least twice more common than the latter. This was in accordance with previous studies [6] showing that mitotic mis-segregation in normal cells consist mainly of sister chromatid non-disjunction (3-1 segregation) and chromatid lagging (2-1 segregation), overall resulting in the generation of a relatively higher proportion of monosomies than trisomies at an approximate 2∶1 ratio. The dual colour FISH approach resulted in a considerably lower prevalence estimate of aneusomy than previous single-probe FISH studies, showing aneusomy rates of 1–20% per chromosome pair [16]–[19], indicating that our approach reduced the rate of false positives. Our estimates were in the same order of magnitude as early studies performed by chromosome banding on lymphocytes and amniocytes [20]. To further validate our approach we performed cytogenetic analyses on multiple primary human fibroblast cultures including F1 and F2 (Tables S1 and S2), all showing an aneusomy prevalence of approximately 1×10^−3^. In contrast, single-probe estimates of the prevalence of aneusomic cells in F1 and F2 were on average nine-fold higher. Taken together, we find that most previous studies have provided overestimates of the prevalence of aneuploidy in non-neoplastic human cells, while a dual colour approach provided a more robust assessment.

The prevalence of aneusomies in F1 and F2 were then compared to Chiron-generated data to deduce the degree of fitness reduction/negative selection for monosomic and trisomic cells compared to diploid cells (Figure 3B). Simulating monoclonal expansion with a normal mis-segregation rate (4×10^−4^), the negative selection resulting from monosomy was found to be on average 28% and that from trisomy 31%, with no significant differences between them (Figure S3). Neither were there any significant differences between the two index chromosomes. To estimate the largest possible margin of error for these calculations, we then also took into account (1) the variability in estimated fibroblast mis-segregation rate of 3.3−4.1×10^−4^ and (2) the fact that variations existed for index chromosomes. This resulted in a span of negative selection of 19% to 50% (reflecting mean ±2 standard deviations). The maximum degree of negative selection in these estimates was invariably far below 100%, implying that elimination of aneuploid cells from a growing normal cell population is typically a process taking several mitotic generations. Because there was no significant difference between trisomy and monosomy, we estimated the overall negative selection against aneusomy using Chiron, resulting in an average of 29% on a relative scale (Figure 3B,E). This means that in a continuously expanding population with 100% probability of re-entering mitosis for diploid cells, a newly generated aneuploid cell would stand a chance of around 49% for proliferative survival up to two generations, but only a 3% chance for surviving up to 10 generations.

### Human Cancer Cells can have Increased Tolerance to Aneuploidy

To make a similar assessment of the degree of cellular fitness reduction resulting from aneuploidy in cancer cells, we used four cancer cell lines (LoVo, DLD1 and SW480 derived from colorectal carcinoma, CRC; WiT49 derived from Wilms tumour, WT) in which we had previously estimated the rate of chromosome mis-segregation [6]. While DLD1 and WiT49 had mis-segregation rates close to those of fibroblasts, LoVo and SW480 showed a high degree of mitotic instability (Table 1). While the stemline of DLD1 was pseudo-diploid, the other lines exhibited substantial aneuploidy with a copy number ≠2 for several chromosomes in the stemline [3], [21]. To estimate the prevalence of aneuploid cells in these lines, FISH analyses with background subtraction were again performed. Because our aim was to study ongoing formation and elimination of aneusomies, the aneusomy index in cancer cell lines was defined as the prevalence of cells with a non-modal copy number for the target chromosome [22]. To be able to monitor possible differences in negative selection between cells changing copy number from disomy and those changing copy number from a pre-existing state of stemline aneusomy, probes were selected to cover chromosomes showing disomy, trisomy and tetrasomy in the stemlines of the various cell lines (Table 1).

**Table 1:** Aneusomy indices and aneuploidy tolerance in cancer cells 1.

	Mode 2	Adjusted AI	Mean s [C]	Min s [C]	Max s [C]
***DLD1***	***“Chromosomally stable”***				
*p = 5x10e^−4^*					
Chr 7	2	0.04	**3%**	3%	4%
Chr 12	2	0.07	**1%**	1%	2%
Chr 18	2	0.02	**4%**	4%	5%
*WiT49*					
*p = 6x10e^−4^*					
Chr 4	2	0.12	**2%**	0.5%	3%
Chr 8	2	0.07	**1%**	0.4%	1%
Chr 15	2	0.11	**2%**	0.5%	3%
Chr 22	2	0.08	**1%**	0.4%	1%
Chr 3	3	0.10	**1%**	0.5%	3%
Chr 6	3	0.11	**2%**	0.4%	3%
Chr 18	3	0.12	**1%**	0.4%	2%
***LoVo***	***“Intermediate”***				
*p = 15x10e^−4^*					
Chr 7	3	0.02	**16%**	15%	17%
Chr 12	3	0.01	**24%**	23%	25%
Chr 18	2	0.03	**6%**	5%	7%
***SW480***	***“Chromosomally unstable”***				
*p = 36x10e^−4^*					
Chr 7	4	0.22	**5%**	4%	5%
Chr 12	2	0.24	**3%**	2%	3%
Chr 18	2	0.32	**2%**	1%	2%

1 Abbreviations: AI, aneusomy index; s, negative selection level;

*p*, mis-segregation rate/chromosome/mitosis.

2 Mode refers to the most common copy number of each chromosome.

**Figure 4:**
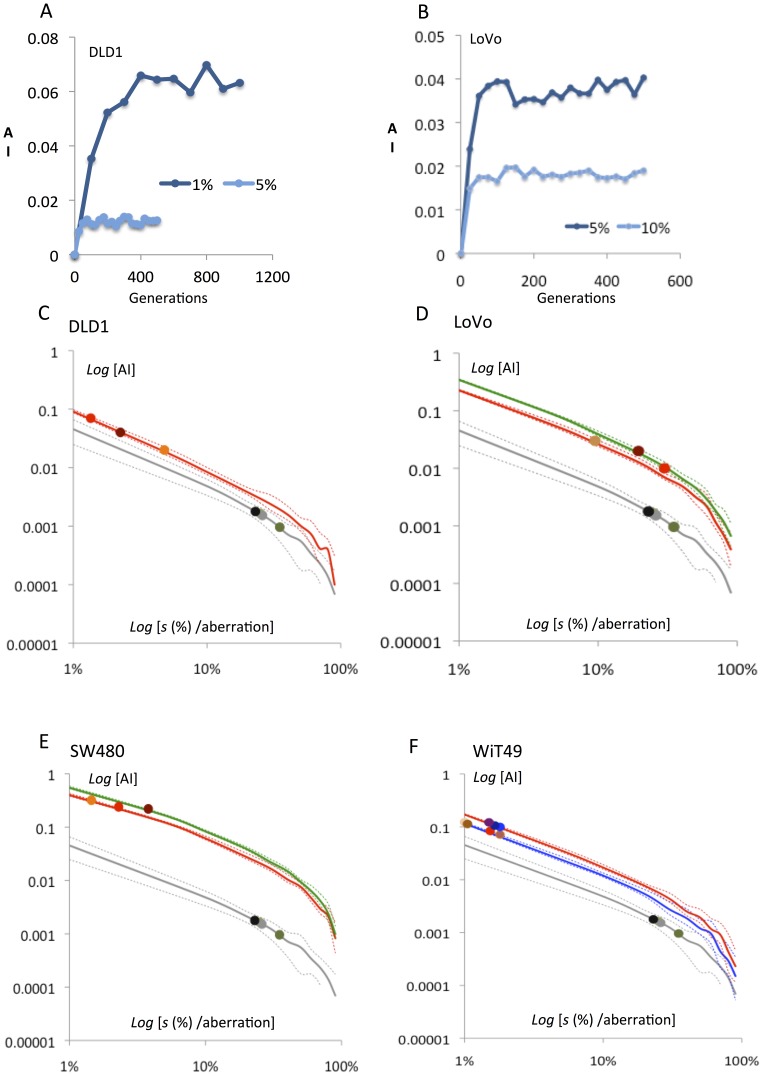
Modelling of aneuploidy rates as a function of negative selection against chromosome changes in cancer cells. (**A–B**) A dynamic equilibrium with respect to aneusomy index (AI) is reached before 500 generations even with very low negative selection pressures in cancer cell populations with a low mis-segregation rate (exemplified by 1% for DLD1 having a near-normal mis-segregation rate). Both plots are from single simulation runs. (**C–F**) **I**n all four analyzed cancer cell lines simulations predicted a near-linear negative relationship between anusomy index (AI) and the degree of negative selection (*s*) on a log-log scale. Full lines indicate mean AI values and broken lines the minimum and maximum values for each simulation of AI for a certain *s*. Grey lines correspond to simulated AI for normal fibroblasts and grey circles indicate AI quantified by FISH for chromosomes 2 and 17 in fibroblasts. Coloured lines correspond to simulated AIs for chromosomes of different modal numbers and coloured circles the FISH-estimated AI values for the analysed chromosomes in the cancer cell lines. Except for one chromosome in LoVo, the negative selection pressures acting on aneusomic cells are lower in the cancer cell lines. The full data calculated from these AI-*s* estimates are presented in Table 1 and summarised in Figure 5.

As expected, the prevalence of cells with non-modal chromosome numbers was considerably (≈10–300 times) higher in the cancer cells lines with elevated chromosomal mis-segregation rates (LoVo and SW480) than in the previously investigated normal cells. However, also in cancer cell populations with a close to normal mis-segregation rate (DLD1 and WiT49), the prevalence of cells with non-modal copy numbers was at least 10 times higher than the average value in fibroblasts (Table 1). This provided indirect evidence for attenuation of aneuploidy-dependent negative selection in cancer cells compared to normal human cells. However, the analysis did not *per se* yield relative values of proliferative survival that could be compared between normal cells and cancer cells. To obtain this, we incorporated the chromosomal mis-segregation rate for each cell line into the Chiron algorithm and used an increasing scale of negative selection against novel aneusomies in a fashion similar to the analysis of fibroblasts. This was done under the assumption that different chromosomes vary little with respect to their individual mis-segregation rates, which is consistent with previous data [4], [6]. Negative selection values for the cancer cell lines were estimated for each chromosome, by matching its aneusomy level to a point of dynamic equilibrium between mis-segregation and elimination by selection (Figure 4). Except for WiT49, the dynamic equilibrium was reached before 500 mitotic generations. Hence equilibrium points from 500 generations were used for estimation of negative selection. WiT49 did not reach equilibrium until ≈1800 generations for negative selection values <0.5% and, therefore, equilibrium points at 2000 generations were used for estimation of negative selection. A monoclonal tumour cell population derived from a continuously regenerating stem cell population can be estimated to have undergone at least 2000 generations before detection [23]. Thus, it is reasonable to assume that WiT49 cells, representing a continuously sub-cultured established cell line, have undergone at least 2000 generations prior to the analyses performed here.

**Figure 5:**
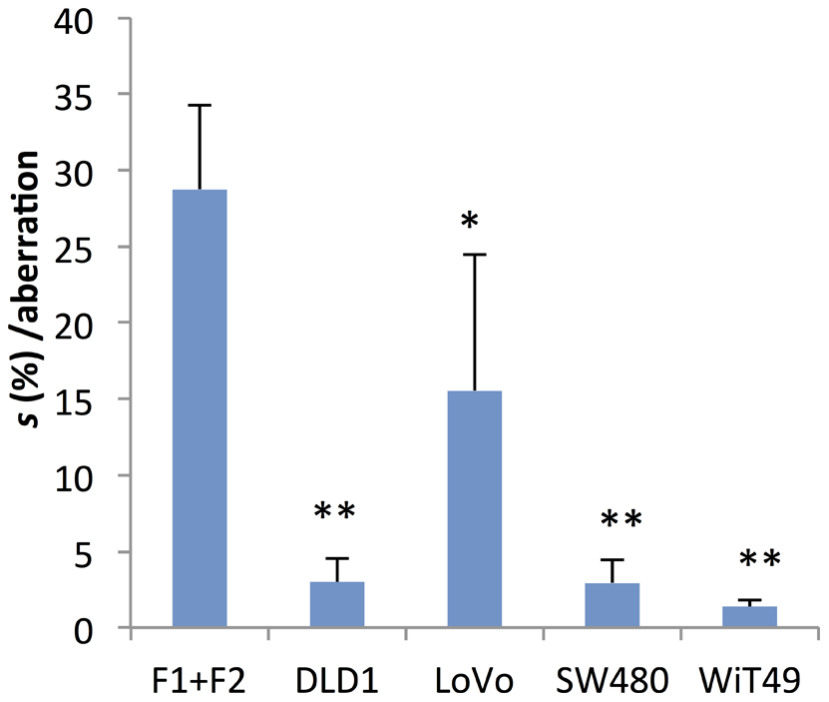
Negative selection acting on aneuploidy in cancer compared to normal cells. Mean aneusomy-dependent negative selection (*s*) estimated by Chiron-simulations. The single star denotes p<0.05 and double stars p<0.001 (Student’s *t*-test) at comparison between each cancer cell line and normal fibroblasts (F1+F2).

Estimation of the negative selection pressures against novel aneusomic cells in the cancer cell populations revealed that LoVo exhibited a larger interchromosomal variation in aneuploidy tolerance than the other three cell lines, with a tendency towards overlap with normal cells (Figures 4 and 5; Table 1). The other three cancer cell populations exhibited less diversity with up to 30-fold lower aneuploidy-dependent negative selection than the normal cells. Taken together, cancer cells showed a mean risk of death/arrest from a newly sustained aneusomy of only 5.8% (p = 0.0008 compared to normal fibroblasts), indicative of aneuploidy tolerance. Notably, this was found not only in WiT49 and SW480, having massive stemline aneuploidy, but also in DLD1 with a pseudo-diploid stemline and aneuploidy present only at the subclonal level [3]. These data indicated that intercellular diversity in chromosome number in cancer cells is dependent not only on an elevated mis-segregation rate of chromosomes, but also on an elevated tolerance to newly acquired aberrations.

### Combined Mitotic Instability and Aneuploidy Tolerance Predict the Scenario of Aneuploidy in Human Cancers

Our results suggested that numerical chromosome changes in cancer may result from a combination of increased mitotic error rate and increased aneuploidy tolerance. In contrast, the vast majority of previous models of aneuploidy in cancer have incorporated only genomic instability caused by elevated mis-segregation rate [3], [4], [6], [7], [22]. To test whether our combined model explained the epidemiological scenario of chromosome aberrations in cancer better than models based on chromosomal instability alone, we analysed published distributions of numerical changes in the tumour types for which our experimental cancer data had been obtained, *i.e*. CRC and WT. Such overall distributions of chromosome aberrations have previously been shown to be highly informative with regard to temporal patterns of clonal evolution and their underlying mechanisms [24]–[27].

**Figure 6:**
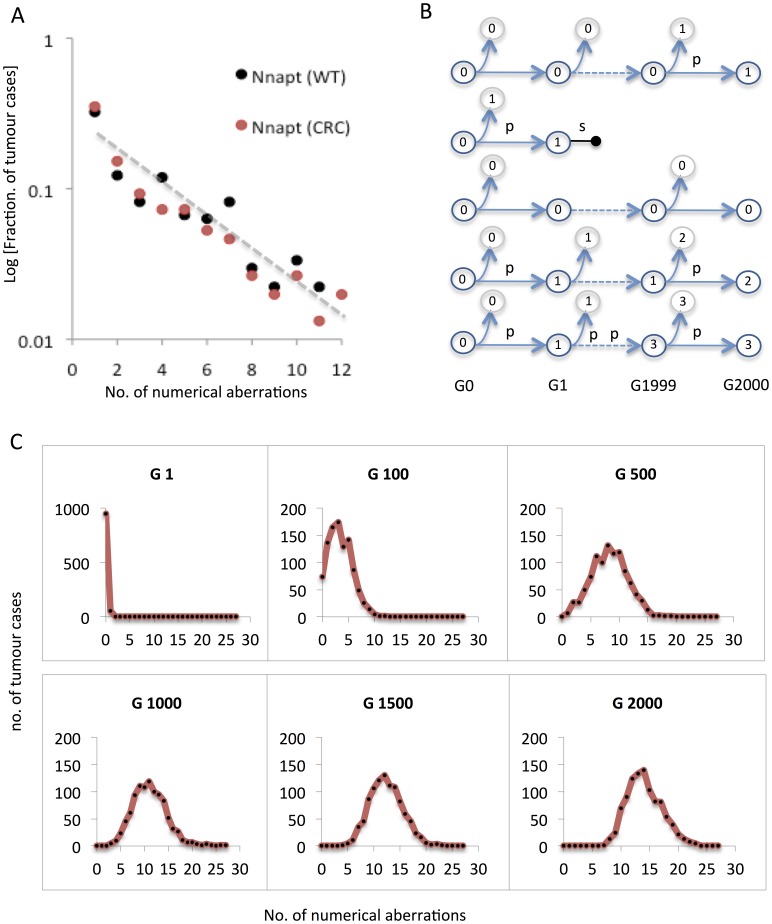
Modelling the distribution of aneuploidy burden in human cancers. (A) Reported cytogenetic data from the Mitelman Database of Chromosome Aberrations and Gene Fusions in Cancer show a log-linear relationship between the relative prevalence and the number of numerical aberrations per tumour (Nnapt), with highly similar distributions for Wilms tumour (WT) and colorectal cancer (CRC). (B) Modelling of a certain number of cancer stemlines arising in the same number of patients. Each stemline is assumed to derive from a diploid cell (having 0 numerical aberrations) and is allowed to proliferate for a maximum of 2000 generations (G), when the overall distribution of numerical aberrations is sampled. Stemlines accumulate numerical aberrations at a certain mis-segregation rate (*p*) and are subject to aneuploidy-dependent selection at a certain degree (*s*), which may in turn result in termination of the stemline (horizontal dumbbell), corresponding to the end of clonal expansion. Because this may result in regression of tumorigenesis at an early stage, cases where stemlines were thus terminated were removed from sampling. (C) Simulated distribution of tumour cases with a certain number of numerical aberrations as the tumour cohort is sampled at generations 1–2000 in a setting where tumours harbour an elevated mis-segregation rate in the absence of negative selection against aneuploid cells (see main text for details). This will result in a binomial-like distribution already after 100 generations, the modal value of which increases with time, in contrast to the actual distribution in human tumours (compare to 6A).

To explore specifically the distribution of numerical aberrations in CRC and WT, cytogenetic data were imported from the Mitelman Database of Chromosome Aberrations and Gene Fusions in Cancer (http://cgap.nci.nih.gov/Chromosomes/Mitelman), comprising 346 and 463 cases with abnormal karyotypes, respectively. After filtering out karyotypes with incomplete or ambiguous cytogenetic information (markers, ring chromosomes, incomplete karyotypes, and diploid cases), there remained karyotypes from 151 CRCs and 269 WTs. Plotting the relative frequencies of tumour cases according to their total number of numerical aberrations revealed a highly similar log-linear distribution (Figure 6A) for both tumour types, where the prevalence of cases with a certain number of aberrations was inversely proportional to the number of aberrations. The distribution was distinctly different from the previously reported overall log-log relationship of aberrations, including structural changes [27]. This indicates that neither of the two theoretical models suggested before for the accumulation of chromosome aberrations in cancer (multiplicative fluctuation and preferential attachment [27]) can explain the pattern of numerical aberrations/aneuploidy in CRC or WT.

**Figure 7:**
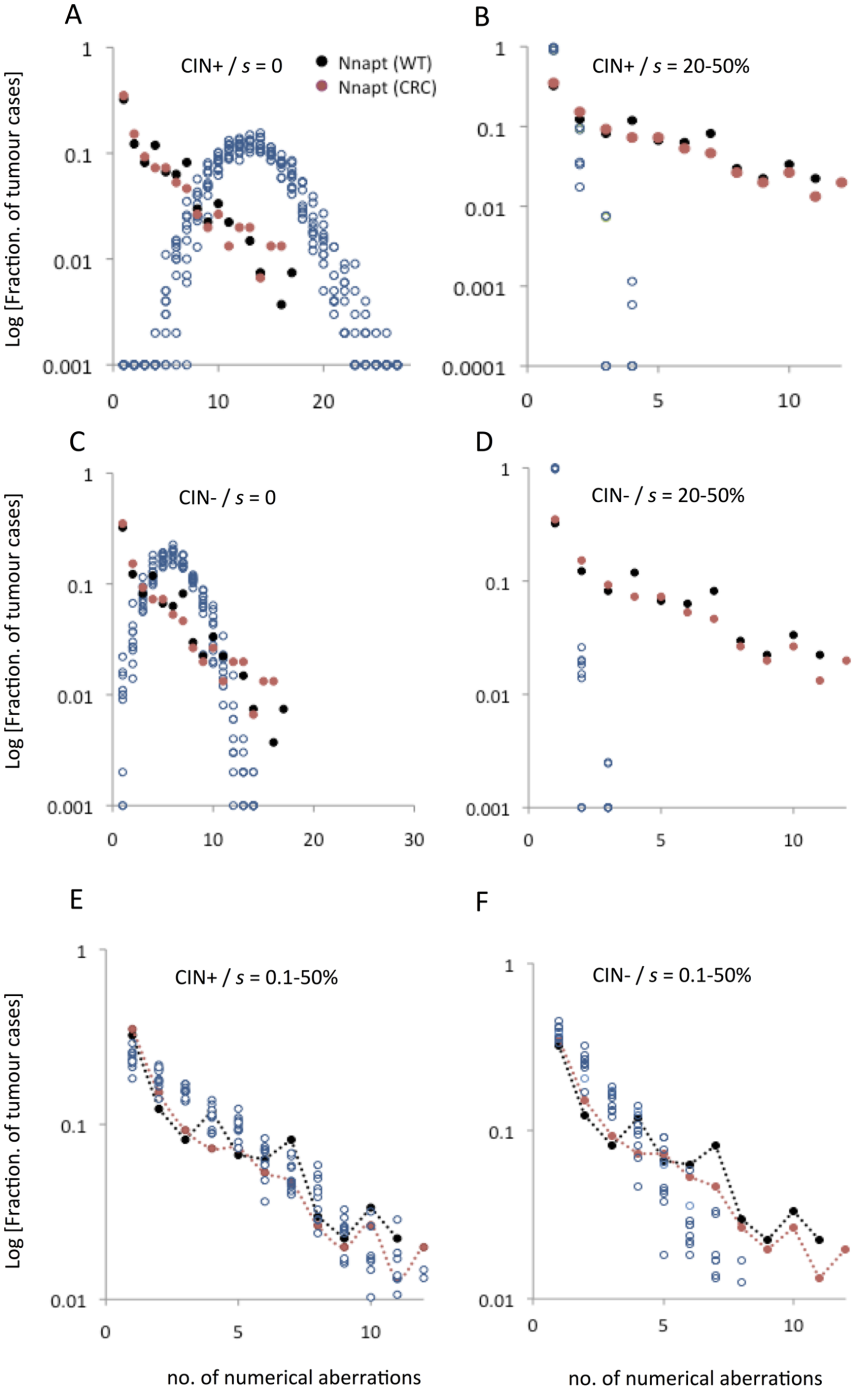
Distribution of numerical aberrations in human cancers predicted by the presence/absence of chromosomal instability and aneuploidy-dependent selection. The expected distributions according to different conditions of chromosomal mis-segregation and selection were predicted by simulations as described in Figure 6. In each graph, the reported data for Wilms tumour (WT, black circles) and colorectal cancer (CRC, red circles) are included for comparison. (A) The expected distribution (open blue circles) of numerical changes in a setting with chromosomal instability (CIN) in the absence of aneuploidy-dependent negative selection (*s*) is binomial-like with a high modal number of aberrations per tumour. All data points from 10 independent simulations were included. (B) A setting with CIN present but with *s* values similar to normal fibroblasts, results in a distribution with far fewer aneusomies than reported in WT and CRC. The same conditions, but with absence of CIN (C and D) result in similar distributions as when CIN is included, but with fewer abnormalities. (E) Attenuated aneusomy-dependent negative selection (*s* set as a span corresponding to the magnitude found in cancer cell lines) and with CIN present predicts a distribution of numerical changes highly similar to reported data. (F) In contrast, attenuated negative selection combined with absence of CIN results in distribution skewed towards fewer numerical aberrations than observed in WT and CRC.

In search of other explanatory models, we set up an algorithm that mimicked the parallel evolution of 500 tumour stemline karyotypes (cancer cases) over 2000 generations [23], each starting with a normal diploid genome, to which different conditions of mitotic instability and selection were applied (Figure 6B). We first tested the standard model of chromosomal instability in cancer, which does not take selection against newly formed aneuploid cells into account, whereas the rate of mitotic chromosome mis-segregation is frequently elevated. Because the mitotic mis-segregation rate is known to vary extensively between tumours, each virtual tumour stemline was assigned a random mis-segregation rate spanning from normal (4×10^−4^/chromosome/mitosis) to the highest value reported (36×10^−4^ [6]). The only selection criterion imposed was obligate extermination of stemlines having obtained nullisomies, based on the fact that tumours with complete absence of material from an autosome have been reported very rarely (http://cgap.nci.nih.gov/Chromosomes/Mitelman). These conditions resulted in a continuous increase of the overall number of chromosome aberrations in the sample population with increasing mitotic generations (Figures 6C and S4A), resulting in a binomial-like distribution with a mode of 15 aberrations after 2000 generations (Figure 7A). Multiple variations in the distribution of mis-segregation rates were also tested, with similar results of a binomial-like distribution.

We then assessed whether a combination of elevated mitotic mis-segregation and an aneuploidy-dependent fitness reduction similar in magnitude to that found in normal cells, would better fit the observed chromosome aberration distributions in CRC and WT. To achieve this, we used the same algorithm as above, but imposed a fitness reduction on aneuploid stemlines that was set to vary randomly between 20% and 50% for different chromosomes. Using these criteria, the overall distribution of numerical changes in the population of stemlines remained stable already after 20 generations and had a modal value of 1 aberration per tumour (Figure 7B). However, no tumour had more than four aberrations under these conditions, again with an overall distribution that was distinctly different from the observed pattern.

Considering that mitotic instability is a phenomenon that has been observed predominantly in established cancer cell lines and that its presence *in vivo* may still be contested, we further tested whether conditions with normal chromosome mis-segregation rate could reproduce the overall observed distribution of numerical aberrations. For this purpose, we performed the two types of simulations described above (with absent and normal selection pressures, respectively) but lowered the mis-segregation rates to normal levels (randomized in the interval 3−5×10^−4^/chromosome/mitosis). These two sets of conditions created distributions of numerical chromosome aberrations (Figure 7C,D) that were highly similar to the corresponding ones with an elevated mis-segregation rate, but with an overall lower number of aberrations, again failing to re-create the pattern of aneuploidy observed in CRC and WT.

We then used the same algorithmic framework and incorporated our findings of a combined elevated mis-segregation rate and aneuploidy tolerance in cancer cells. For this purpose, the mis-segregation rates of the 500 virtual stemlines (cancer cases) were again randomized between 4 and 36×10^−4^, while the fitness reduction on aneuploid cells was varied on a three-tiered scale based on our experimental data from the highest value found in normal cells (50%) to low (1%) to very low (0.1%). Under these conditions, the distribution of chromosome aberrations in the 500 virtual stemlines reached a stable state at approximately 500 generations, after which it showed a log-linear distribution with a mode of 1 and a range from 1–12, faithfully recreating the pattern of numerical aberrations observed in CRC and WT (Figures 7E and S4B). We also evaluated the consequences of setting the negative selection pressure to a series of identical constant values in the range of 0.1–10% for all stemlines (cancer cases), but this again failed to replicate the observed data (Figure S4C). So did a number of simulations in which various levels of constant negative selection rates were combined with various spans or variable chromosomal mis-segregation rates. Taken together, this indicated that a scenario of inter-tumour variability with respect to aneuploidy tolerance best explains the published cytogenetic data. Finally, we also tested the outcome of a similarly attenuated negative selection combined with a normal mis-segregation rate. Although this also resulted in a log-linear distribution, it was skewed towards fewer number of aberrations than observed in CRC and WT, with no single case showing >10 changes (Figure 7F).

Taken together, our attempts to recreate the general statistical features of reported numerical aberrations in two different tumour types, showed that a model where elevated mitotic mis-segregation rate is combined with a variable degree of attenuated negative selection against aneuploidy better reflects the observed data than more conservative models based on (1) elevated mis-segregation combined with no aneuploidy intolerance, (2) elevated mis-segregation combined with a normal (high) aneuploidy intolerance, (3) normal (low) mis-segregation rate combined with no aneuploidy intolerance, (4) normal (low) mis-segregation rate combined with normal (high) aneuploidy intolerance, (5) elevated mitotic mis-segregation rate combined with a degree of attenuated aneuploidy intolerance that is the same over different tumour cases, and (6) normal (low) mis-segregation rate combined with attenuated aneuploidy intolerance. This indicated that our findings of a variably increased tolerance to aneuploidy in cancer cells could be applied to explain cancer genomic data better than previous models of genomic instability.

### Aneuploidy Tolerance Allows Clonal Expansion of Cells with Aneuploidy

**Figure 8:**
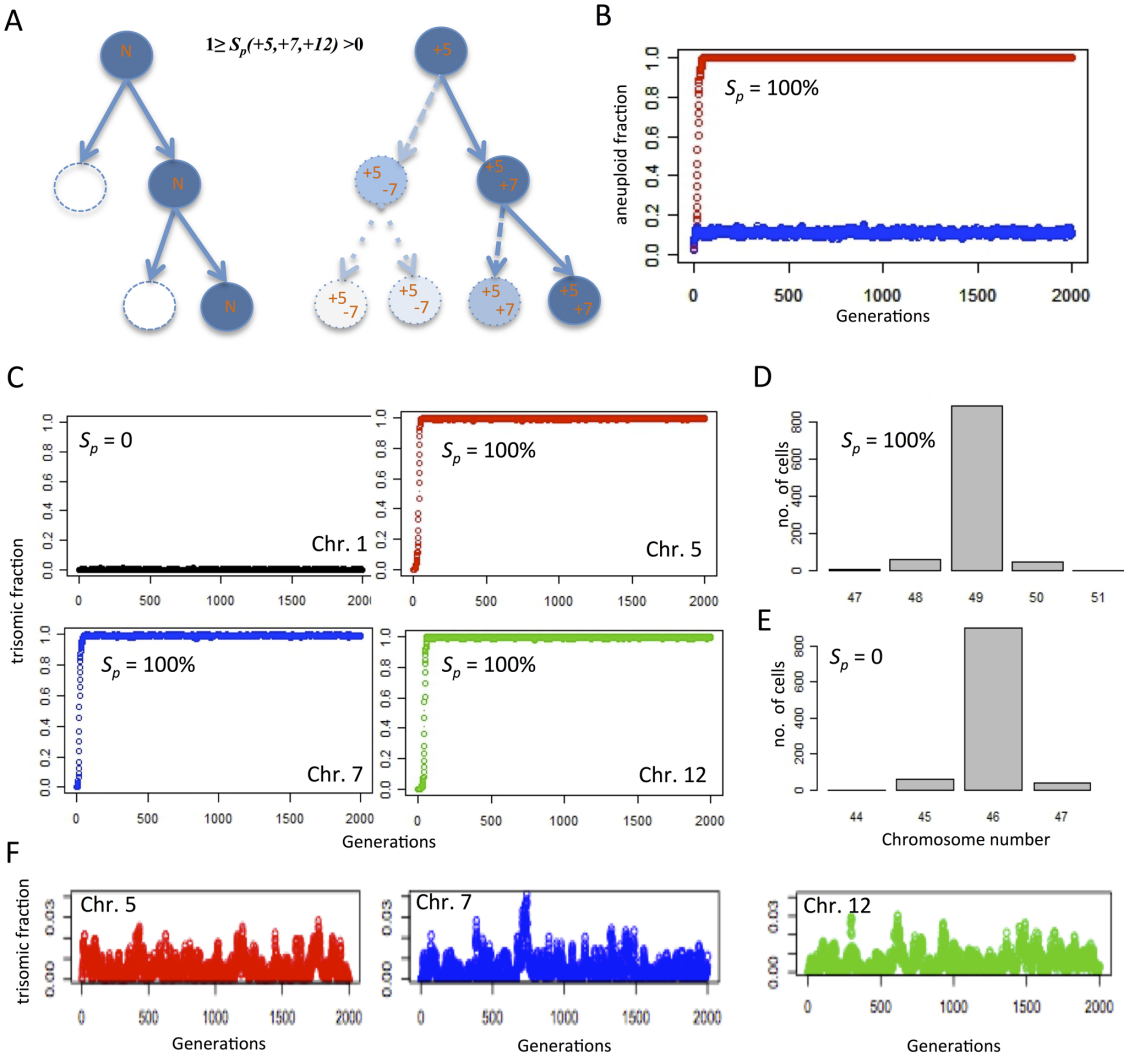
Reverse engineering *in silico* of the LoVo stemline genome by introducing positive selection. (A) The LoVo stemline with trisomies of chromosomes 5, 7 and 12 was recreated by modelling clonal expansion from a normal diploid (N) state where aneuploid cells were subject to negative selection (*S*_n_) at the magnitude measured in LoVo, with the exception of cells with one or more of the trisomies +5, +7, +12. Such cells were subjected to a variable degree of positive selection (*S*_p_), *i.e.* a higher probability of undergoing mitotic proliferation than diploid cells. For simplicity, diploid cells were given an average probability of 50% for proliferative survival, resulting in one daughter cell on overage for each mother cell (left). Cells having acquired +5/+7/+12 were set to generate on average >1 daughter cell, with the maximum survival benefit for cells having all three trisomies (right). *S*_p_ was set in relation to the proliferation of normal cells with *S*_p_ = 100% equalling the generation of 2 daughter cells per mother cell with all three trisomies. Each of +5/+7/+12 were coupled to an identical degree of positive selection i.e. 1/3 *S*_p_ for each. Aneusomies were acquired through mis-segregation at the rate measured in LoVo. (B) The prevalence of aneuploid cells reaches a stable, high level in the population (1,000 cells) with 100% positive selection for +5/+7/+12 (red plot), while a parallel proliferation in absence of positive selection retains a diploid genome in the majority of cells (blue plot). (C) In the same simulation of positive selection, the prevalence of cells with each of +5/+7/+12 increase rapidly; chromosome 1, used as an internal control, remains disomic. (D) When the prevalence of aneuploidy has reached a stable state the modal number has shifted to 49 as expected with positive selection for +5/+7/+12, while it remains diploid (E) in the absence of positive selection for trisomies. (F) Using the same parameters as in B–D but introducing *S*_n_ = 30% for all aneusomies including +5/+7/+12 prevents expansion of aneuploid clones. Instead a state of mutation/selection balance is reached where trisomic cells never reach a higher prevalence than 4%.

To validate further our model of combined chromosomal instability and attenuated negative selection against aneuploidy, we reverse-engineered *in silico* the stemline of the LoVo cancer cell line. The majority of cells in this population exhibit concurrent trisomies for chromosomes 5, 7 and 12, its stemline karyotype being 49,XY,t(2;12)(q23;p13),+5,+7,+12, i(15)(q10). The stable presence of these trisomies in multiple reports on LoVo sub-lines, despite its elevated chromosomal mis-segregation rate [3], [28]–[31], indicates that they are important for continued proliferation. In accordance with this, we constructed a virtual, homogeneously diploid population of pre-tumour cells that were allowed to proliferate under conditions where cells having obtained one or more of these trisomes were subjected to positive selection (Figure 8A). Although there is ample evidence from *in vitro* and *in vivo* animal models that aneuploidy may confer an increased cellular fitness under specific conditions [32], [33], data regarding the magnitude of positive selection based on aneuploidy in cancer cells is scarce. Our model was therefore constructed according to the most simple arbitrary cumulative model: cells having acquired all three trisomies were awarded twice the probability of proliferative survival compared to cells not having any of +5/+7/+12, while cells having one of the trisomies was awarded 1/3, and cells having two of them awarded 2/3 of this survival advantage. In the algorithm, this was formalised by setting the average probability of proliferative survival for diploid cells to 50%. Hence any euploid 46,XY cell would on average give rise to one daughter cell, while a cell having the karyotype 49,XY,+5,+7,+12 would give rise to an average of two daughters. We then incorporated the known LoVo chromosomal mis-segregation rate (15×10^−4^/chromosome/mitosis; Table 1) as well as its average estimated fitness reduction from aneuploidy (22% risk of proliferative death for changes other than +5/+7/+12; Table 1) into the model and allowed this virtual cell population to grow for up to 2000 generations.

Under these conditions, there was invariably (100/100 simulations) a clonal expansion leading up to a majority (mean 88%) stemline population with 49,XY,+5,+7,+12 (Figure 8B–D). Removing the positive selection for cells with any of +5/+7/+12 from the algorithm resulted in 0 cells with two or all trisomes, and a mean of 0.1% of cells having any one of them, a level only slightly higher than those of trisomies for other chromosomes (Figure 8E). In this set of first simulations, there was no negative selection imposed on cells having obtained +5, +7 or +12 as opposed to other numerical aberrations for which a fitness reduction was imposed. To test whether increased aneuploidy tolerance was a necessary condition for clonal expansion in this model, we re-instated a normal relative risk (30%, approximated from 29% in fibroblast data) of proliferative death for cells having sustained any alteration in chromosome number while keeping positive selection for +5/+7/+12 as above. Just like the situation where there was no positive selection pressure, this resulted in a failure of aneuploid cells to dominate the population, with a maximum of 3% of cells having the karyotype 49,XY,+5,+7,+12 when multiple consecutive simulations were monitored over 2000 generations (Figure 8F). Hence, positive selection acting to promote proliferative survival for cells with a particular pattern of aneuploidy failed to generate a predominance of aneuploid cells when aneuploidy intolerance was not concurrently reduced, even though trisomic cells appeared at higher prevalence levels than in the absence of positive selection (compared to chromosome 1 in panel C).

### Dominance of Positive Over Negative Selection Leads to Rapid Clonal Expansion also in Chromosomally Stable Cells

**Figure 9:**
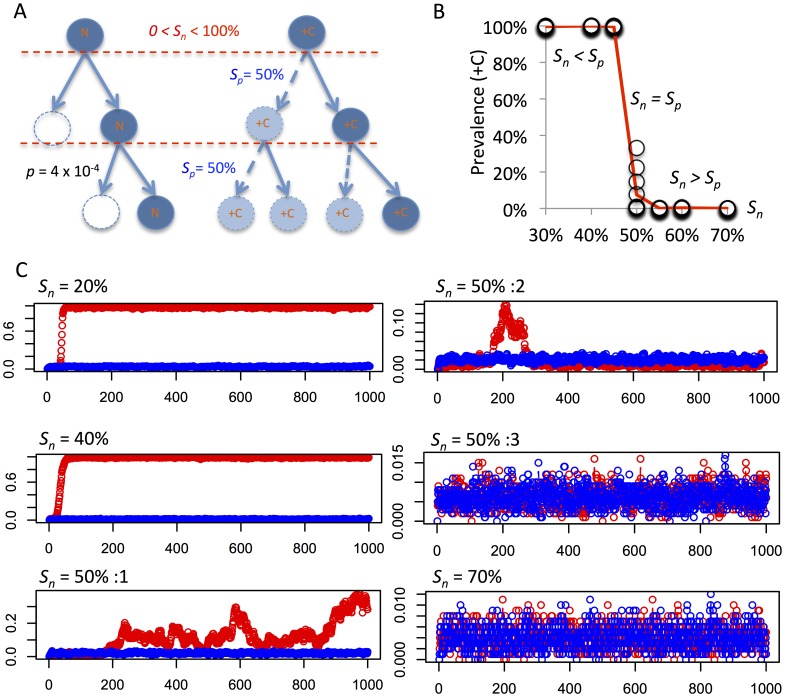
Clonal expansion in a context of normal chromosome mis-segregation. (A) Evaluation of clonal expansion of cells with trisomy for a single chromosome (+C), set up under similar conditions as described in Figure 8 but with a mis-segregation rate similar to normal fibroblasts (*p* = 4×10^−4^), a constant rate of positive selection (*S_p_*) of 50%, and a variable degree of negative selection against aneuploidy (*S_n_*). Parallel simulations were performed with a control population with equal conditions except for an absence of positive selection. (B, C) A clonal expansion resulting in dominance of cells with +C (prevalence >99%) after 1000 generations is observed when *S_p_*> *S_n_*, while *S_p_* = *S_n_* results in variable prevalence of cells with +C, including expansion up to a prevalence of 33% (*S_n_* = 50%:1 in C), expansion followed by regression (*S_n_* = 50%:2 in C), and a lack of clonal expansion (*S_n_* = 50%:3 in C). Each circle in B corresponds to a single run of simulations, with 10 runs per level of *S_n_*, while the red line corresponds to mean results. Red plots in C correspond to prevalence of +C cells under positive selection and blue plots reflect the results of parallel control simulations with *S_p_* = 0; x axes denote mitotic generations.

Clonal expansion should in theory occur under all conditions where the fitness gain of a certain chromosome alteration outweighs the negative selection against aneuploidy. This infers that clonal expansion of aneuploid cells should be able to occur in conditions also with a normal chromosomal mis-segregation rate if the balance between positive and negative selection acting on aneuploidy was perturbed. To model this situation, we set up a small virtual cell population (1000 cells) that were allowed to proliferate under a constant mis-segregation rate similar to that in fibroblasts (Figure 9A). A constant, high positive selection of 50% for all cells obtaining trisomy for a certain chromosome (+C) was introduced, computed in an identical fashion to the LoVo simulations except for the lower positive selection for a single trisomy in that model. The system was then tested with a span of different negative selection levels against aneuploidy. As expected, simulations where positive selection predominated (*S_p_*> *S_n_*) resulted in a dominance (>99% prevalence) of trisomic cells with an extra copy of chromosome C, after less than 100 generations (Figure 9B and C), while the opposite condition (*S_p_*< *S_n_*) failed to generate clonal expansion of +C cells. Notably, simulations with identical positive and negative selection pressure (*S_p_* = *S_n_*) resulted in an unpredictable system, with the prevalence of +C cells ranging from 0 to 33%, providing a correlate to cytogenetic sub-clonality. In all, these simulations showed that even under chromosomally stable conditions such as in non-neoplastic tissue, and in very small populations, an expansion of aneuploid cells can occur if the aneuploidy in question confers a net positive fitness effect.

## Discussion

### Aneuploidy Tolerance in Neoplastic and Non-neoplastic Cells

The present study provides evidence from multiple human cell systems, including primary cells, that a certain degree of aneuploidy intolerance exists in non-neoplastic human cells, and that this intolerance can be attenuated in cancer to allow long-term clonal expansion of cells with an elevated chromosomal mis-segregation rate. It thereby corroborates previous scarce investigations suggesting that specific aneuploidy tolerance mechanisms exist in human cells [14], [34]. The relatively low aneuploidy tolerance we found in normal cells helps explaining why novel chromosome copy number aberrations can be formed at a fairly high rate in human somatic tissues under non-neoplastic conditions, although the prevalence of aneuploidy remains low. Our findings could also potentially explain how a high prevalence of chromosomal imbalances in mosaic form can be present at early stage embryos without a corresponding high prevalence of miscarriages, foetal deaths, or live-born children with chromosomal anomalies [35], [36]. Indeed, it has been reported that 91% of early human embryos analysed in the process of pre-implantation genetic diagnosis show chromosomal imbalances in at least one blastomere, while the *in vitro* fertilization success rate after this procedure is above 20% [37]. By showing that a combination of aneuploidy tolerance and an increased chromosomal mis-segregation rate better explains published genomic cancer data than does genomic instability alone, the present study also suggests that a modified cellular response to aneuploidy must be taken into account when addressing mechanisms behind genomic landscapes in cancer. Finally, our investigation provides a framework of computer models/algorithms dealing with the combined effects of mitotic instability and selection in human cells. The data derived from these models may provide a starting point for quantitative comparisons of aneuploidy tolerance between different human tissues and tumour types.

### Weaknesses and Pitfalls

The present study is to a large extent based on *in silico* analyses into which experimental data have been integrated. It must be questioned to what extent our models in fact predict true biological phenomena. We have attempted to amend this by basing our simulations on thoroughly background-filtered empirical data on aneuploidy prevalence in normal human cells and cancer cells. A remaining caveat for the present study is that *in vivo* conditions are extrapolated from *in vitro* data, although obtained after only short-time culture. In normal cells, our estimates of the prevalence of aneuploid cells were considerably lower than previous investigations, which were performed with a single-probe approach. This indicates that background filtration is essential for estimating aneuploidy in cell populations where it is a rare phenomenon. However, it can still be questioned to what extent our aneuploidy data obtained from fibroblasts reflect the situation in the cell types from which most cancers arise. Other studies that have estimated the mis-segregation rate of chromosomes in non-tumour cells have found data highly similar to our results in a span of different cell types [4], [13], [14]. Similar to the present study, it has also been reported that induction of a high mis-segregation rate in non-cancer established cell lines will fail to induce a corresponding increase in the prevalence of aneuploid cells [14], [34], indicative of aneuploidy intolerance in normal diploid cells. Our findings are thus in line with the few previous studies addressing the complex issue of chromosomal instability and aneuploidy tolerance.

The present study indicates that aneuploidy tolerance may be at least as important as proper mitotic control for keeping the rate of aneuploid cells low in healthy human tissues. Nevertheless, there may be notable exceptions to this situation, which are not addressed by the present study. First, the aneuploidy tolerance may vary extensively between different human tissues, cell types, and tumour types. For example, a high rate of chromosomal mosaicism is known to be present in the human placenta [38], brain [39]–[41] and liver [42]. Furthermore, normal human pluripotent stem cell lines have recently been reported to harbour a relatively high proportion of cells with non-clonal aneuploidies [43]. However, besides the findings in placenta, most of these studies were performed with single-probe FISH and may report rates of aneuploidy including an unknown proportion of false positives. On the other hand, it is known from decades of clinical cytogenetic analysis, primarily of blood, bone marrow, and amniocytes, that clonal or sub-clonal aneuploidy is exceedingly rare in dividing human cells from these tissue types [20]. Second, the cellular response to aneuploidy can to some extent be expected to depend on which chromosome is present at an abnormal copy number, as evidenced by the relatively high rate of sex chromosome loss with age [16], [18], [19]. We chose two autosomes (2 and 17) of different size as index chromosomes and found no difference in the degree of tolerance for their respective aneusomies. Our findings are consistent with previous studies showing no difference for differently sized chromosomes with respect to somatic autosomal aneuploidy in human lymphocytes, endothelial cells or fibroblasts [16], [18], [19]. On the other hand, our assumption of a fairly constant level of selection against newly formed aneuploid cells should also be seen in the light of a recent major study showing that aneuploidy increases with age, when comparisons over several decades are made [44]. Whether this age-dependent increase results from a slow attenuation of the aneuploidy response over time in adult individuals, or if it is caused by an elevated rate of mis-segregation with age is presently unclear.

For cancer cells, our findings of relative aneuploidy tolerance were obtained from established cancer cell lines. Using primary human tumour cells would require a reliable methodology for differentiating neoplastic cells from accompanying non-neoplastic tumour cells at genetic *in situ* analyses. The validity of our aneuploidy tolerance estimates from cell lines were tested against genomic data from 420 human tumours and were found to predict their distribution of whole chromosome changes better than previous models. We would therefore claim that our data at least provide a reasonable order of magnitude for aneuploidy intolerance in cancer cells. In contrast, our final models including positive selection for aneuploidy in cancer (Figures 8 and 9) were based entirely on *in silico* algorithms and our predictions in this respect should be regarded as a set of theoretically based hypotheses that mandate experimental validation. For example, it should be theoretically possible for a certain genetic change to confer a sufficiently high selective advantage to break through even the high negative selection barrier present in normal/intolerant cells, and consequently dominate the population (Figure 9). However, studies of the effects of point mutations in cancer has indicated that their relative selective advantages may be small [45]. For whole-chromosome changes, that cannot possibly have a uniform positive selection advantage on the gene level, breaking through the negative selection barrier by virtue of positive selection alone therefore appears unlikely, although not impossible as shown by our final simulation of clonal expansion of aneuploid cells in a population with normal mis-segregation rate. Although several model systems have shown that some specific aneuploidies may be selected for under certain conditions [11], [32], [46], experimental evidence relating directly to the quantitative balance between positive and negative selection of a certain chromosome change in human cells is almost totally lacking, which makes it difficult to correlate our findings to experimental data. A further weakness of our models of selection for or against aneuploidy is that they do not take into account interaction between different types of aberrations that may influence selection in a non-additive manner. Finally, they do not account for exceptional circumstances where mis-segregation rates vary considerably between chromosomes or between different sub-clones of a tumour [3], [47].

### Implications for Cancer Genomics

Our findings indicate that the occurrence of aneuploidy in cancer cells is best explained as a combination of increased mitotic error rate and an elevated tolerance to newly sustained whole chromosome alterations. However, the relative contributions of these two aspects varied greatly among cancer cell populations. In DLD1 and WiT49, the relatively high rate of aneuploidy could to a large extent be attributed to tolerance because mis-segregation rates were close to normal in both these cell lines. Furthermore, reverse engineering *in silico* of clonal expansion in a cell population with a normal mis-segregation rate showed that the occurrence of an aneuploid stemline was critically dependent on the net fitness effect of aneuploidy rather than on chromosomal instability. Even though our findings indicate that aneuploidy intolerance is a stronger limiting factor than chromosomal instability for generation of clonal aneuploidy, it does not exclude that aneuploidy tolerance is selected in cancer for reasons unrelated to the formation of an aneuploid stemline. The latter case is illustrated by our finding of aneuploidy tolerance also in DLD1, a cancer cell line with a diploid stemline having only a few segmental imbalances. This suggests that aneuploidy tolerance may in some instances precede aneuploidy in cancer development. Taken together, our study shows a quantifiable presence of aneuploidy-tolerance in human cancer cells, which may signal the presence of aneuploidy-tolerating mutations in neoplastic cells akin to those identified in lower eukaryotes [12], [48]. Along the same lines, our study indirectly supports recent data suggesting that compounds interfering with pathways of specific importance for survival of aneuploidy-tolerant cells could serve as novel anti-cancer agents [48].

## Materials and Methods

### Ethics Statement

The study of somatic genetic variation in anonymised biobanked tissue samples from the Departments of Clinical Genetics and Pathology, Lund University, was approved (ref. no 2012-405) by the ethics review board of Lund University. Written consent was obtained from donors to the biobank. Samples were taken as part of routine diagnostic procedures, and only cells remaining after such procedures had been finalised were used for research purposes. The sampling process was documented in the filing system of Lund University Hospital.

### Cell Culture and FISH Analysis of Fibroblasts

Fibroblasts were obtained from the NIGMS Human Genetic Cell Repository/Corriell Institute for Medical Research (Camden, NJ). The fibroblasts (GM05399, GM00500B) were from healthy males, aged 1 (F1) and 10 (F2) respectively. The colorectal cancer cell lines were purchased from ATCC (LGC Standards, Teddington UK), while WiT49 was donated by Prof. Herman Yeger, Department of Paediatric Laboratory Medicine, The Hospital for Sick Children, Toronto, Canada [49]. Karyotypically normal anonymised control lymphocyte and primary fibroblast samples were obtained from the biobank at the Department of Clinical Genetics, Lund University. Cell culture, harvest, chromosome preparation, and FISH were according to standard methods [50]. In brief, cells were cultured in DMEM F-12 with antibiotics and 10% fetal bovine serum, harvested and fixed in methanol:acetic acid (3∶1), after which slides were pepsinised, dehydrated, formalin-fixed, and used for FISH. To increase the precision of the copy number analysis, each chromosome was detected with two probes with different spectral signatures, one targeting centromeric repeats (C) and one targeting a single copy region located to a chromosome arm (A). Chromosome 2 was detected by the probes LSI NMYC SG/CEP 2 SO and chromosome 17 by the probes LSI 17q SO/CEP 17 SA (Abbott Molecular Inc, Des Plaines, IL). In total, 12,433 interphase fibroblast nuclei were analyzed and all signal configurations scored. Images were acquired using a video camera coupled to an Axioplan 2 fluorescence microscope (Carl Zeiss, Jena, Germany). Image analysis was done using the CytoVision system (Applied Imaging, Newcastle Upon Tyne, UK).

For each chromosome, an overall aneusomy rate (*OAR*) in F1 and F2 was determined as the prevalence of nuclei exhibiting a non-disomic pattern (*i.e.* not 2C +2A). Tetraploidisation (recognised as enlarged nuclei with a concordant 4C +4A pattern) and other variants of polyploidisation were not included in the *OAR* estimates because it is known to occur physiologically as well as over time in cell culture [18], [19]. The frequency of nuclei with different types of discordant signal patterns for the arm and centromere probes *e.g.* 1C+2A and 3C+2A (discordant false aneusomy, *DFA*) was used to estimate the combined frequency of cells with failure of hybridisation fidelity and cells with confounding structural chromosomal rearrangements. These *DFA* values were also used to calculate the expected prevalence of nuclei simulating true aneusomies by concurrent unspecific hybridisation of two probes or structural rearrangements, (concordant false aneusomy, *CFA*; Figure S3). A corrected aneuploidy rate (*CAR*) was calculated as *CAR = OAR – DFA – CFA* and was assumed to be the estimate closest reflecting the true prevalence of aneusomic cells. Underestimation of aneusomy was deemed highly unlikely as (1) the frequency of cells with loss of a single signal was similar for both probe types, (2) the prevalence of cells with 0 signals was <0.1%, (3) a hypothetical systematic underestimation of trisomies by hybridisation failure of one probe would be counterbalanced by an over-estimation of monosomies.

### Chiron Software Construction and Simulations of Selection in Normal Cells

The Chiron software was implemented in C++ using the QT and Boost.Random libraries. In short the simulation algorithm can be described as follows (Figure S1): An object containing a list of 23 integers represents a cell in this model. At the start of every simulation a cell was created by setting each of the items in the list to a user-defined copy number representing the copy number of the individual chromosomes. For diploid cells this number was 2, as X- and Y-chromosomes were treated as a homologous pair. This initial cell was then added to a list of cells called the current generation, and then the simulation proceeded using the algorithm described in Figure S1.

After the end of a simulation, the program exported the karyotypes of all cells in the sampled generations. Since every cell gives rise to two daughter cells, the number of cells in the n:th generation would be 2^n^ cells. This would rapidly lead to a number of cells in each generation that would be too large to handle for standard memory hardware, even for low numbers of n. To amend this, the program randomly sampled 10,000 cells from each generation to create the subsequent generation if the total population was larger than this number. Multiple control simulations where the population size became stationary at different cell numbers (10,000–100,000) did not produce significantly different end results.

In order to assess the aneuploidy-dependent negative selection value that corresponded to a certain prevalence of aneusomic cells (aneusomy index, AI), the following procedure was applied: for each cell line, simulations were performed with different selection levels per aneusomy type (monosomy, trisomy, overall aneusomy). From these data, AIs corresponding to the different selection levels were plotted and compared to the experimentally observed value of AI, to determine the selection level that corresponded to each level of aneusomy, given the experimentally found frequency of non-disjunction. The margin of prediction error for the selection levels was estimated by shifting the simulated curves by +2 and −2 standard deviations.

### FISH Analysis and Simulations of Selection in Cancer Cells

DLD1, LoVo, and SW480 were obtained from the American Type Culture Collection through LCG Standards (Borås, Sweden), while WiT49 was obtained from Dr. Herman Yeger, Developmental & Stem Cell Biology, University of Toronto, Canada. Cells were cultured according to instructions given by the providers and FISH analyses performed as for fibroblasts. Because these cell lines show aneuploidy rates considerably higher than normal cells, FISH analyses were first performed with centromeric probes for chromosomes with different modal numbers in the stemline (Abbott Molecular Inc), to determine an unfiltered frequency of cells with non-modal copy numbers (*OAR*) for each chromosome. This was followed by background reduction by subtracting the *OAR* for each chromosome with *OARs* obtained with the same centromeric probes on normal peripheral blood lymphocytes. Control experiments on DLD1 showed that this approach revealed similar *CAR* values after background reduction as the two-colour approach used for fibroblasts (data not shown). Levels of aneuploidy-dependent negative selection were determined by Chiron as for fibroblast data.

### Reverse Engineering of Cancer Karyotypes

Recreation of the LoVo karyotype was performed by creating a virtual cell population in an R 2.11.0 GUI 1.33 Leopard build 64-bit environment, with each cell having a diploid (2×23) genome at generation 0. Each type of simulations was first initiated with 100,000 cells and then scaled down to the minimum population size where results remained stable. To save calculation time, we then performed repeated simulations at this possible smallest possible sample size consisting of 1,000 cells if not otherwise specified. Copy numbers of each chromosome were allowed to evolve in discrete generational steps through mis-segregation in a fashion identical to previous simulations, with a mis-segregation rate of 15×10^−4^/chromosome/mitosis if not otherwise specified. The cell population was kept constant by assuming an overall average reproductive rate of 1 daughter per mother cell. Cells that aquired 0 copies of any chromosome were invariably excluded from subsequent generations and replaced with a copy of another randomly selected cell in the next generation. Any cell selected to undergo aneuploidy-dependent proliferative death according to the given probability for this to happen was replaced in a similar fashion. Cells selected to give rise to 2 daughters by having an aneuploidy pattern subjected to positive selection was copied together with its specific mis-segregation rate to replace another, randomly selected cell from its own generation. Simulations were terminated after 2000 generations or until a clone derived from a single founder cell hade overtaken the entire population (1,000/1,000 cells). Creation of a virtual cancer stem line with a single trisomy (+C) through positive selection was performed in a fashion identical to reverse engineering of LoVo but with a chromosome mis-segregation of 4×10^−4^/chromosome/mitosis and with a constant positive selection for cells with +C of 50% compared to cells with a normal chromosome C copy number. The degree of negative selection was varied between 20% and 70%. All simulations were terminated after 1000 generations. For each level of negative selection, 10 simulations were performed. R- based simulation algorithms will be provided through email by the corresponding author on request.

## Supporting Information

Figure S1 **Chiron algorithm.** Schematic representation of the algorithm underlying the Chiron software for simulating monoclonal growth.
(TIFF)

Figure S2 **Aneusomy assessment by dual colour fluorescence in situ hybridization (FISH).** Each chromosome was labelled with a centromeric (green) and a locus-specific probe for one of the chromosome arms (red). This allowed recognition of cells that would be scored as false positives (monosomic or trisomic) by a single-probe approach (upper panel). The frequency of false positives in turn makes it possible to calculate the expected number of double false positives (erroneous extra signals from both probes; lower panel).
(TIFF)

Figure S3 **Prevalence of cells with trisomies and monosomies by Chiron simulations.** Trisomy (TI) and monosomy (MI) index at dynamic equilibrium for different strengths of aneuploidy-dependent negative selection (*s*) in a cell population with normal mis-segregation rate (4×10^−4^/chromosome/mitosis). Broken lines correspond to assessments of *s* from FISH-estimated TI (green) and MI (red) values.
(TIFF)

Figure S4 **Simulated distribution of numerical aberrations in human cancers.** The evolution of 500 independent tumour stemlines (cases) were simulated by introducing different combinations of chromosomal instability (CIN) and aneuploidy-dependent negative selection (*s*). (A) Simulation of two independent populations of stemlines (Pop1 and Pop2) in a setting where CIN is present and *s* = 0. The maximum number of aberrations (Max A) increased over consecutive generations (upper panels), resulting in binomial-like distributions of numerical aberrations at the end of simulation (lower panel). (B) CIN combined with attenuated negative selection in a proportion of tumours (see article text for details) resulted in a stabilization of the maximum number of aberrations at an early stage and a distribution of aberrations dominated by stemlines with relatively few changes. See main text for details. (C) Applying a constant value of *s* instead of an empirically based span fails to re-create the reported distributions from cytogenetic data, indicating that *s* is variable among human tumours with some having high *s* values corresponding to those in non-tumour cells. Mis-segregation rates were randomized in the span 4–36×10^−4^ as for simulations in Figures 6 and 7. See main text and Legend to Figure 7 for details.
(TIFF)

Table S1 **Estimation of AI in fibroblasts by single- versus dual-color FISH.**
(DOCX)

Table S2 **Estimation of AI in normal cells by chromosome banding.**
(DOCX)

Protocol S1 **Chiron software user instructions.** The software is available for download in one version for PC/Windows and one for Mac/OS users.
(DOCX)

Software S1 **Chiron for Mac OS.**
(ZIP)

Software S2 **Chiron for PC/Windows.**
(ZIP)

## References

[1] Artandi SE, Chang S, Lee SL, Alson S, Gottlieb GJ, et al (2000) Telomere dysfunction promotes non-reciprocal translocations and epithelial cancers in mice. Nature 406: 641–645. 10949306 10.1038/35020592

[2] Gisselsson D, Jonson T, Petersen A, Strombeck B, Dal Cin P, et al (2001) Telomere dysfunction triggers extensive DNA fragmentation and evolution of complex chromosome abnormalities in human malignant tumors. Proc Natl Acad Sci U S A 98: 12683–12688. 11675499 10.1073/pnas.211357798 PMC60114

[3] Stewenius Y, Gorunova L, Jonson T, Larsson N, Hoglund M, et al (2005) Structural and numerical chromosome changes in colon cancer develop through telomere-mediated anaphase bridges, not through mitotic multipolarity. Proc Natl Acad Sci U S A 102: 5541–5546. 15809428 10.1073/pnas.0408454102 PMC556242

[4] Ganem NJ, Godinho SA, Pellman D (2009) A mechanism linking extra centrosomes to chromosomal instability. Nature 460: 278–282. 19506557 10.1038/nature08136 PMC2743290

[5] Silkworth WT, Nardi IK, Scholl LM, Cimini D (2009) Multipolar spindle pole coalescence is a major source of kinetochore mis-attachment and chromosome mis-segregation in cancer cells. PLoS One 4: e6564. 19668340 10.1371/journal.pone.0006564 PMC2719800

[6] Gisselsson D, Jin Y, Lindgren D, Persson J, Gisselsson L, et al (2010) Generation of trisomies in cancer cells by multipolar mitosis and incomplete cytokinesis. Proc Natl Acad Sci U S A 107: 20489–20493. 21059955 10.1073/pnas.1006829107 PMC2996656

[7] Janssen A, van der Burg M, Szuhai K, Kops GJ, Medema RH (2011) Chromosome segregation errors as a cause of DNA damage and structural chromosome aberrations. Science 333: 1895–1898. 21960636 10.1126/science.1210214

[8] Gisselsson D, Pettersson L, Hoglund M, Heidenblad M, Gorunova L, et al (2000) Chromosomal breakage-fusion-bridge events cause genetic intratumor heterogeneity. Proc Natl Acad Sci U S A 97: 5357–5362. 10805796 10.1073/pnas.090013497 PMC25833

[9] Lord CJ, Ashworth A (2012) The DNA damage response and cancer therapy. Nature 481: 287–294. 22258607 10.1038/nature10760

[10] Sheltzer JM, Amon A (2011) The aneuploidy paradox: costs and benefits of an incorrect karyotype. Trends Genet 27: 446–453. 21872963 10.1016/j.tig.2011.07.003 PMC3197822

[11] Williams BR, Prabhu VR, Hunter KE, Glazier CM, Whittaker CA, et al (2008) Aneuploidy affects proliferation and spontaneous immortalization in mammalian cells. Science 322: 703–709. 18974345 10.1126/science.1160058 PMC2701511

[12] Torres EM, Dephoure N, Panneerselvam A, Tucker CM, Whittaker CA, et al (2010) Identification of aneuploidy-tolerating mutations. Cell 143: 71–83. 20850176 10.1016/j.cell.2010.08.038 PMC2993244

[13] Cimini D, Tanzarella C, Degrassi F (1999) Differences in malsegregation rates obtained by scoring ana-telophases or binucleate cells. Mutagenesis 14: 563–568. 10567031 10.1093/mutage/14.6.563

[14] Thompson SL, Compton DA (2008) Examining the link between chromosomal instability and aneuploidy in human cells. J Cell Biol 180: 665–672. 18283116 10.1083/jcb.200712029 PMC2265570

[15] Neurath P, DeRemer K, Bell B, Jarvik, Kato T (1970) Chromosome loss compared with chromosome size, age and sex of subjects. Nature 225: 281–282. 5409983 10.1038/225281a0

[16] Guttenbach M, Koschorz B, Bernthaler U, Grimm T, Schmid M (1995) Sex chromosome loss and aging: in situ hybridization studies on human interphase nuclei. Am J Hum Genet 57: 1143–1150. 7485166 PMC1801353

[17] Mukherjee AB, Thomas S (1997) A longitudinal study of human age-related chromosomal analysis in skin fibroblasts. Exp Cell Res 235: 161–169. 9281365 10.1006/excr.1997.3673

[18] Mukherjee AB, Thomas S, Schmitt E (1995) Chromosomal analysis in young vs. senescent human fibroblasts by fluorescence in situ hybridization: a selection hypothesis. Mech Ageing Dev 80: 11–23. 7564557 10.1016/0047-6374(94)01544-v

[19] Aviv H, Khan MY, Skurnick J, Okuda K, Kimura M, et al (2001) Age dependent aneuploidy and telomere length of the human vascular endothelium. Atherosclerosis 159: 281–287. 11730807 10.1016/s0021-9150(01)00506-8

[20] Petersson H, Mitelman F (1985) Nonrandom de novo chromosome aberrations in human lymphocytes and amniotic cells. Hereditas 102: 33–38. 3988540 10.1111/j.1601-5223.1985.tb00462.x

[21] Stewenius Y, Jin Y, Ora I, de Kraker J, Bras J, et al (2007) Defective chromosome segregation and telomere dysfunction in aggressive Wilms’ tumors. Clin Cancer Res 13: 6593–6602. 18006759 10.1158/1078-0432.CCR-07-1081

[22] Lengauer C, Kinzler KW, Vogelstein B (1997) Genetic instability in colorectal cancers. Nature 386: 623–627. 9121588 10.1038/386623a0

[23] Tomlinson I, Bodmer W (1999) Selection, the mutation rate and cancer: ensuring that the tail does not wag the dog. Nat Med 5: 11–12. 9883827 10.1038/4687

[24] Hoglund M, Gisselsson D, Hansen GB, Sall T, Mitelman F (2002) Multivariate analysis of chromosomal imbalances in breast cancer delineates cytogenetic pathways and reveals complex relationships among imbalances. Cancer Res 62: 2675–2680. 11980667

[25] Hoglund M, Gisselsson D, Hansen GB, Sall T, Mitelman F, et al (2002) Dissecting karyotypic patterns in colorectal tumors: two distinct but overlapping pathways in the adenoma-carcinoma transition. Cancer Res 62: 5939–5946. 12384560

[26] Hoglund M, Frigyesi A, Sall T, Gisselsson D, Mitelman F (2005) Statistical behavior of complex cancer karyotypes. Genes Chromosomes Cancer 42: 327–341. 15645488 10.1002/gcc.20143

[27] Frigyesi A, Gisselsson D, Mitelman F, Hoglund M (2003) Power law distribution of chromosome aberrations in cancer. Cancer Res 63: 7094–7097. 14612501

[28] Melcher R, Koehler S, Steinlein C, Schmid M, Mueller CR, et al (2002) Spectral karyotype analysis of colon cancer cell lines of the tumor suppressor and mutator pathway. Cytogenet Genome Res 98: 22–28. 12584437 10.1159/000068544

[29] Masramon L, Ribas M, Cifuentes P, Arribas R, Garcia F, et al (2000) Cytogenetic characterization of two colon cell lines by using conventional G-banding, comparative genomic hybridization, and whole chromosome painting. Cancer Genet Cytogenet 121: 17–21. 10958935 10.1016/s0165-4608(00)00219-3

[30] Drewinko B, Romsdahl MM, Yang LY, Ahearn MJ, Trujillo JM (1976) Establishment of a human carcinoembryonic antigen-producing colon adenocarcinoma cell line. Cancer Res 36: 467–475. 1260746

[31] Tsushimi T, Noshima S, Oga A, Esato K, Sasaki K (2001) DNA amplification and chromosomal translocations are accompanied by chromosomal instability: analysis of seven human colon cancer cell lines by comparative genomic hybridization and spectral karyotyping. Cancer Genet Cytogenet 126: 34–38. 11343776 10.1016/s0165-4608(00)00391-5

[32] Pavelka N, Rancati G, Zhu J, Bradford WD, Saraf A, et al (2010) Aneuploidy confers quantitative proteome changes and phenotypic variation in budding yeast. Nature 468: 321–325. 20962780 10.1038/nature09529 PMC2978756

[33] Duncan AW, Hanlon Newell AE, Bi W, Finegold MJ, Olson SB, et al (2012) Aneuploidy as a mechanism for stress-induced liver adaptation. J Clin Invest 122: 3307–3315. 22863619 10.1172/JCI64026 PMC3428097

[34] Thompson SL, Compton DA (2010) Proliferation of aneuploid human cells is limited by a p53-dependent mechanism. J Cell Biol 188: 369–381. 20123995 10.1083/jcb.200905057 PMC2819684

[35] Li M, DeUgarte CM, Surrey M, Danzer H, DeCherney A, et al (2005) Fluorescence in situ hybridization reanalysis of day-6 human blastocysts diagnosed with aneuploidy on day 3. Fertil Steril 84: 1395–1400. 16275234 10.1016/j.fertnstert.2005.04.068

[36] Munne S, Velilla E, Colls P, Garcia Bermudez M, Vemuri MC, et al (2005) Self-correction of chromosomally abnormal embryos in culture and implications for stem cell production. Fertil Steril 84: 1328–1334. 16275225 10.1016/j.fertnstert.2005.06.025

[37] Vanneste E, Voet T, Le Caignec C, Ampe M, Konings P, et al (2009) Chromosome instability is common in human cleavage-stage embryos. Nat Med 15: 577–583. 19396175 10.1038/nm.1924

[38] Yuen RK, Robinson WP (2011) Review: A high capacity of the human placenta for genetic and epigenetic variation: implications for assessing pregnancy outcome. Placenta 32 Suppl 2S136–141. 21281965 10.1016/j.placenta.2011.01.003

[39] Yurov YB, Iourov IY, Vorsanova SG, Liehr T, Kolotii AD, et al (2007) Aneuploidy and confined chromosomal mosaicism in the developing human brain. PLoS One 2: e558. 17593959 10.1371/journal.pone.0000558 PMC1891435

[40] Westra JW, Rivera RR, Bushman DM, Yung YC, Peterson SE, et al (2010) Neuronal DNA content variation (DCV) with regional and individual differences in the human brain. J Comp Neurol 518: 3981–4000. 20737596 10.1002/cne.22436 PMC2932632

[41] Iourov IY, Vorsanova SG, Liehr T, Yurov YB (2009) Aneuploidy in the normal, Alzheimer’s disease and ataxia-telangiectasia brain: differential expression and pathological meaning. Neurobiol Dis 34: 212–220. 19344645 10.1016/j.nbd.2009.01.003

[42] Duncan AW, Hanlon Newell AE, Smith L, Wilson EM, Olson SB, et al (2012) Frequent aneuploidy among normal human hepatocytes. Gastroenterology 142: 25–28. 22057114 10.1053/j.gastro.2011.10.029 PMC3244538

[43] Peterson SE, Westra JW, Rehen SK, Young H, Bushman DM, et al (2011) Normal human pluripotent stem cell lines exhibit pervasive mosaic aneuploidy. PLoS One 6: e23018. 21857983 10.1371/journal.pone.0023018 PMC3156708

[44] Jacobs KB, Yeager M, Zhou W, Wacholder S, Wang Z, et al (2012) Detectable clonal mosaicism and its relationship to aging and cancer. Nat Genet 44: 651–658. 22561519 10.1038/ng.2270 PMC3372921

[45] Beerenwinkel N, Antal T, Dingli D, Traulsen A, Kinzler KW, et al (2007) Genetic progression and the waiting time to cancer. PLoS Comput Biol 3: e225. 17997597 10.1371/journal.pcbi.0030225 PMC2065895

[46] Upender MB, Habermann JK, McShane LM, Korn EL, Barrett JC, et al (2004) Chromosome transfer induced aneuploidy results in complex dysregulation of the cellular transcriptome in immortalized and cancer cells. Cancer Res 64: 6941–6949. 15466185 10.1158/0008-5472.CAN-04-0474 PMC4772432

[47] Zhu J, Pavelka N, Bradford WD, Rancati G, Li R (2012) Karyotypic determinants of chromosome instability in aneuploid budding yeast. PLoS Genet 8: e1002719. 22615582 10.1371/journal.pgen.1002719 PMC3355078

[48] Tang YC, Williams BR, Siegel JJ, Amon A (2011) Identification of aneuploidy-selective antiproliferation compounds. Cell 144: 499–512. 21315436 10.1016/j.cell.2011.01.017 PMC3532042

[49] Alami J, Williams BR, Yeger H (2003) Derivation and characterization of a Wilms’ tumour cell line, WiT 49. Int J Cancer 107: 365–374. 14506735 10.1002/ijc.11429

[50] Gisselsson D (2001) Refined characterisation of chromosome aberrations in tumours by multicolour banding and electronic mapping resources. Methods Cell Sci 23: 23–28. 11741141

