# Supplementary figures and images for: Elevated Tolerance to Aneuploidy in Cancer Cells: Estimating the Fitness Effects of Chromosome Number Alterations by In Silico Modelling of Somatic Genome Evolution
Source: PLoS One. 2013 Jul 24;8(7):e70445. doi: 10.1371/journal.pone.0070445 (PMC3722120; doi:10.1371/journal.pone.0070445)

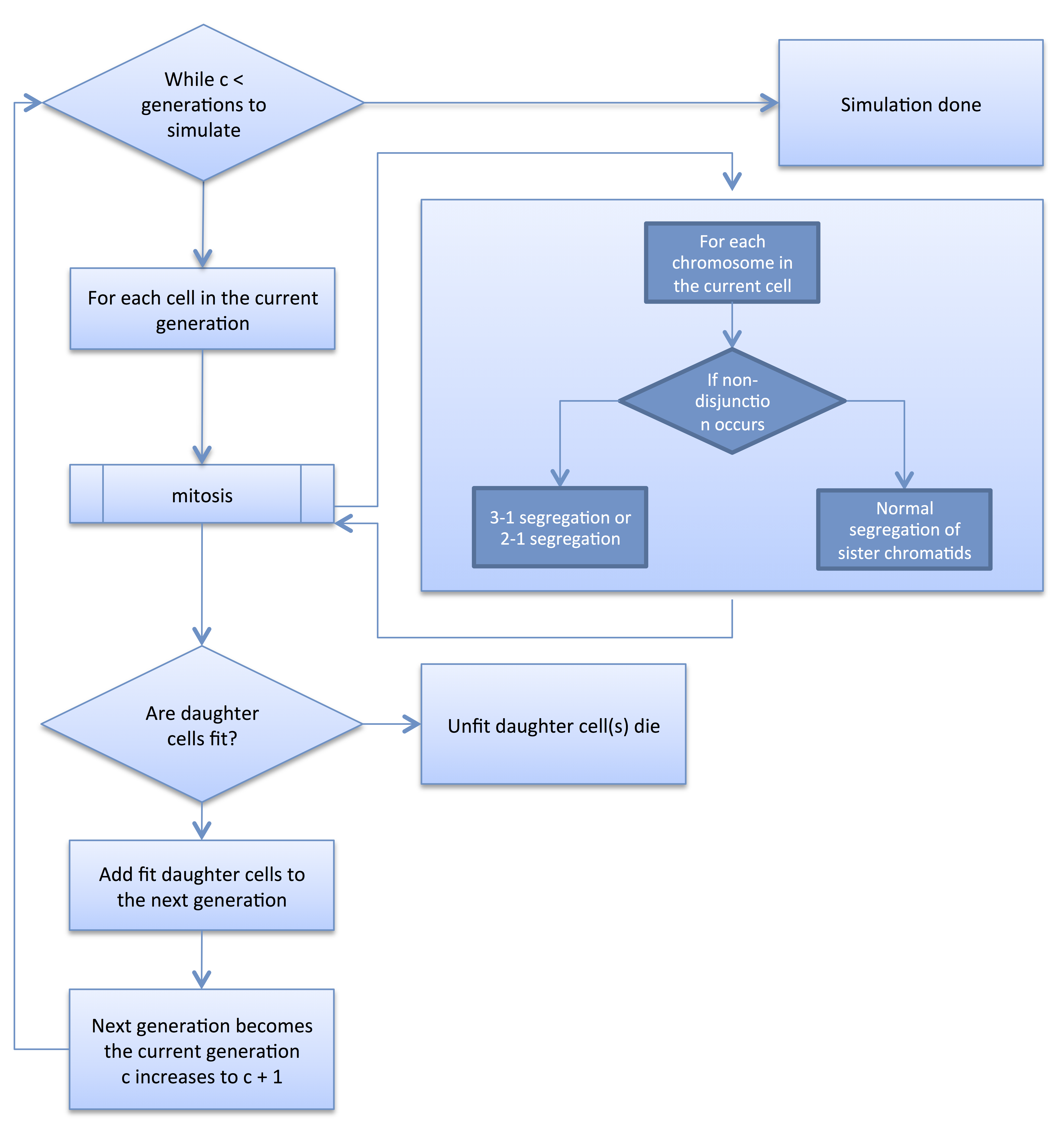

Supplement: Figure S1 — Chiron algorithm. Schematic representation of the algorithm underlying the Chiron software for simulating monoclonal growth. (TIFF) [file pone.0070445.s001.tif]

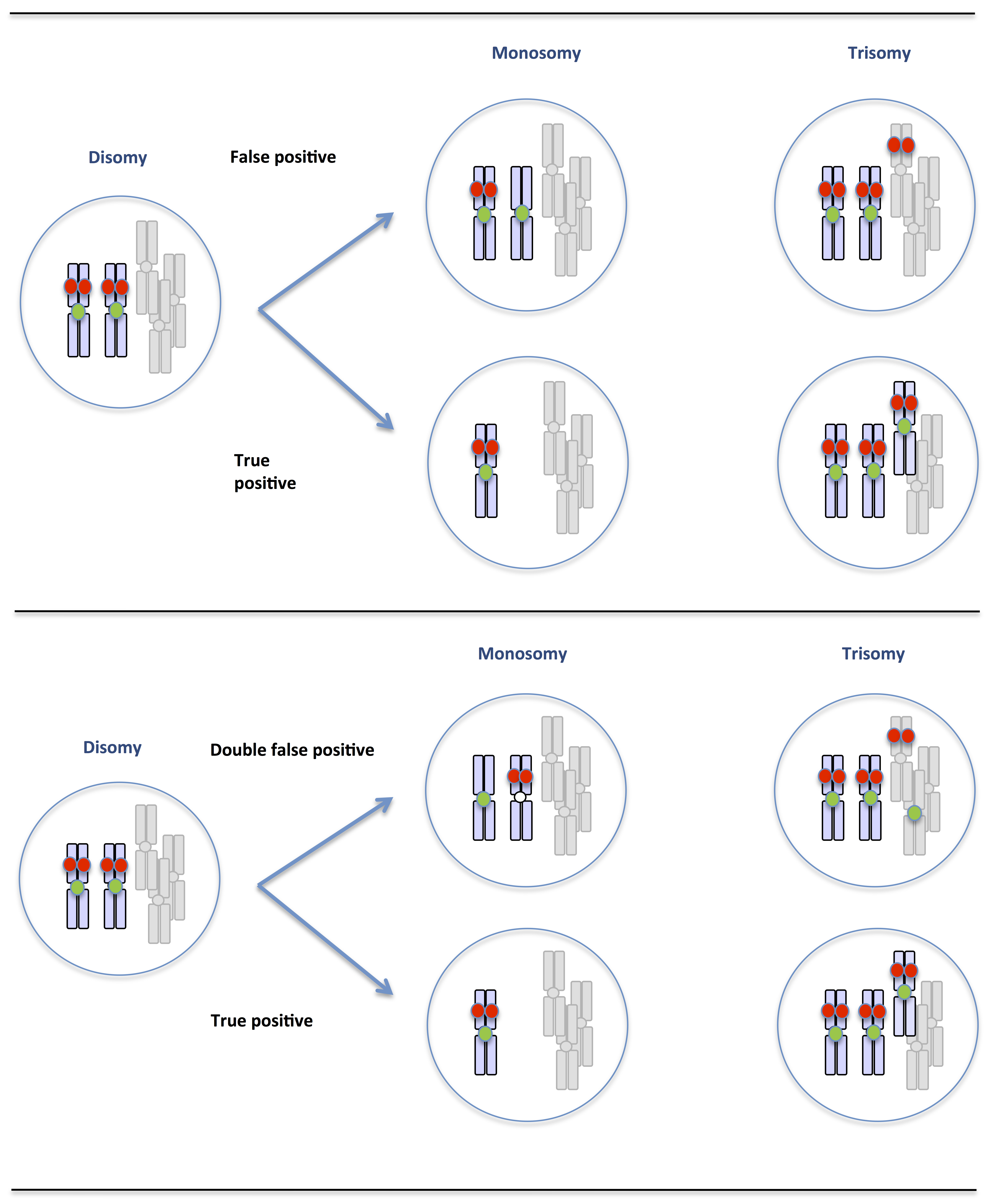

Supplement: Figure S2 — Aneusomy assessment by dual colour fluorescence in situ hybridization (FISH). Each chromosome was labelled with a centromeric (green) and a locus-specific probe for one of the chromosome arms (red). This allowed recognition of cells that would be scored as false positives (monosomic or trisomic) by a single-probe approach (upper panel). The frequency of false positives in turn makes it possible to calculate the expected number of double false positives (erroneous extra signals from both probes; lower panel). (TIFF) [file pone.0070445.s002.tif]

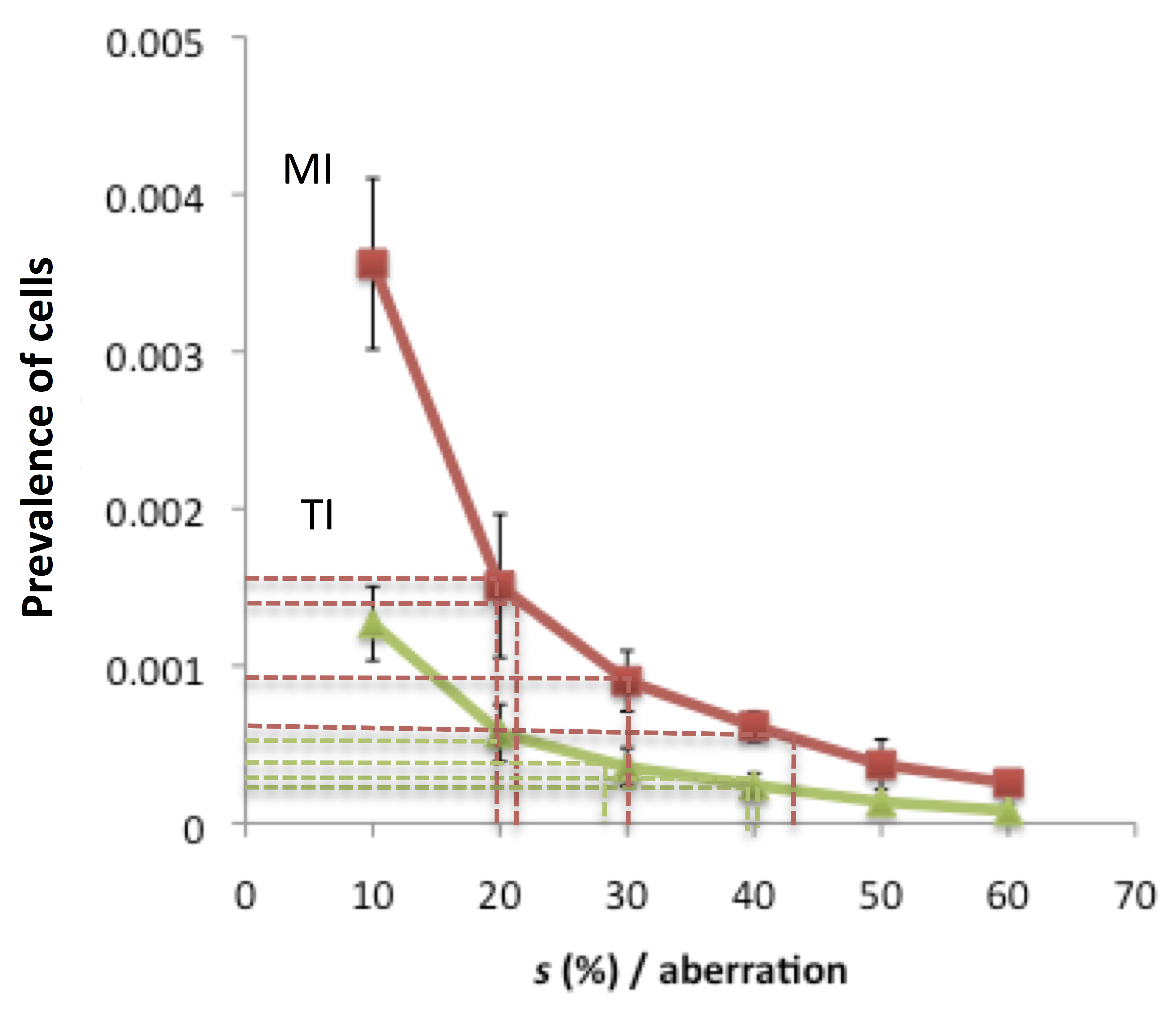

Supplement: Figure S3 — Prevalence of cells with trisomies and monosomies by Chiron simulations. Trisomy (TI) and monosomy (MI) index at dynamic equilibrium for different strengths of aneuploidy-dependent negative selection (s) in a cell population with normal mis-segregation rate (4×10^−4^/chromosome/mitosis). Broken lines correspond to assessments of s from FISH-estimated TI (green) and MI (red) values. (TIFF) [file pone.0070445.s003.tif]
