# Supplementary material for: Elevated Tolerance to Aneuploidy in Cancer Cells: Estimating the Fitness Effects of Chromosome Number Alterations by In Silico Modelling of Somatic Genome Evolution
Source: PLoS One. 2013 Jul 24;8(7):e70445. doi: 10.1371/journal.pone.0070445 (PMC3722120; doi:10.1371/journal.pone.0070445)
Supplement: Table S1 — Estimation of AI in fibroblasts by single- versus dual-color FISH. (DOCX) [file pone.0070445.s005.docx]

**Table S1.** Estimation of AI in fibroblasts by single- versus dual-color FISH

| **Cell-chr-probe^1^** | **AI^2^ single colour** | **AI^2^ dual colour** | **Single / Dual** |
| --- | --- | --- | --- |
|  |  |  |  |
| F1-chr2-R | 5.23E-03 | 9.60E-04 | 5 |
| F1-chr2-G | 5.56E-03 |  | 6 |
| F1-chr17-R | 1.26E-02 | 1.60E-03 | 8 |
| F1-chr17-B | 2.37E-02 |  | 15 |
| F2-chr2-R | 7.71E-03 | 1.50E-03 | 5 |
| F2-chr2-G | 1.57E-02 |  | 10 |
| F2-chr17-R | 1.10E-02 | 1.80E-03 | 6 |
| F2-chr17-B | 2.04E-02 |  | 11 |
|  |  |  |  |
| **Mean** | **12.7E-03** | **1.47E-03** | 9 |
|  |  |  |  |

^1^ R, G, and B = red (spectrum orange) green (spectrum green), and blue (spectrum aqua) labeled probes

^2^ AI = aneusomy index per pair of homologous chromosomes
