# Supplementary material for: Elevated Tolerance to Aneuploidy in Cancer Cells: Estimating the Fitness Effects of Chromosome Number Alterations by In Silico Modelling of Somatic Genome Evolution
Source: PLoS One. 2013 Jul 24;8(7):e70445. doi: 10.1371/journal.pone.0070445 (PMC3722120; doi:10.1371/journal.pone.0070445)
Supplement: Table S2 — Estimation of AI in normal cells by chromosome banding. (DOCX) [file pone.0070445.s006.docx]

**Table S2.** Estimation of AI in normal cells by chromosome banding

| **Cell type^1^** | **No. of cells analysed** | **No. of aneuploid cells** | **Percentage aneuploid cells** | **AI^2^** |
| --- | --- | --- | --- | --- |
|  |  |  |  |  |
| Lymphocytes | 17,545 | 244 | 1.66% | 7.24E-04 |
| Amniotic fluid cells | 17,262 | 220 | 1.46% | 6.35E-04 |
| Fibroblasts | 240 | 4 | 1.67% | 7.25E-04 |
|  |  |  |  |  |
| **Mean** |  |  | 1.60% | **0.69E-03** |

^1^ Data for lymphocytes and amniotic fluid cells were obtained from Peterson and Mitelman (1985) Hereditas 102: 33-38; fibroblast data were obtained from 14 karyotypically normal skin biopsy samples

^2^ AI = aneusomy index per pair of homologous chromosomes
