# Supplementary material for: Elevated Tolerance to Aneuploidy in Cancer Cells: Estimating the Fitness Effects of Chromosome Number Alterations by In Silico Modelling of Somatic Genome Evolution
Source: PLoS One. 2013 Jul 24;8(7):e70445. doi: 10.1371/journal.pone.0070445 (PMC3722120; doi:10.1371/journal.pone.0070445)
Supplement: Protocol S1 — Chiron software user instructions. The software is available for download in one version for PC/Windows and one for Mac/OS users. (DOCX) [file pone.0070445.s007.docx]

**Protocol S1**

**User instructions to the Chiron software.**

The software is enclosed for download in separate versions for MS Windows and Mac OS.

**Introduction**

Chiron is a software for exploring how cells accumulate numerical chromosome aberrations over time due to chromosome mis-segregation under various negative selection pressures.

**Parameters**

The parameters that can be set by the user are the following:

| **Parameter name** | **Example** | **Explanation** |
| --- | --- | --- |
| Number of generations | 1,50,100 | A comma-separated list of generations that you want to sample from the simulation |
| Non-disjunction probability | 5e-4 | The per chromosome per mitosis rate of mis-segregation |
| Number of runs | 10 | Number of times the simulation will repeat in parallel runs |
| Copy number | 2 | The copy number of all chromosomes in the progenitor cell. |
| Specify Karyotype |  | The user can specify a karyotype with different copy numbers for different chromosomes in the progenitor cell. |
| Use chromatid loss | ON/OFF | Whether the simulation should include losses of single chromatids (2-1 division). |

**Selection**

The different types of fitness conditions that can be imposed on the cells are:

- ANY – A condition acting on all chromosomes in all cells that have the specified copy number.
- ANY_NOT – A condition acting on all chromosomes in all cells that do not have the specified copy number

There are also conditions acting on single chromosomes, which have the form **CHR_N** and **CHR_N_NOT** where N stands for the chromosome copy number, for instance CHR_3, and CHR_5_NOT. Each fitness condition is composed of three values, first, the type, *i.e.* ANY, ANY_NOT etc, the copy number that the condition acts on, and the probability of cell death for that condition. For instance, to specify a negative selection level (probability of cell death) of 0.1 for every trisomy 8 the following

Fitness condition would be used:

| **Chromosome** | **Copy number** | **P(death)** |
| --- | --- | --- |
| CHR_8 | 3 | 0.1 |
|  |  |  |

**Generated data**

The saved data is in the form of .csv-files (comma-separated files), one for each generation for each repetition of the simulation. All the files for each generation are saved in a subdirectory. Each row in these .csv-files has the total number of chromosomes of that cell, as well as the copy number for all individual chromosomes. From these raw data files various metrics can be calculated. The most common metric used are aneusomy index (the percentage of cells carrying a copy number of a specific chromosome different from the one specified in the progenitor cell) or aneuploidy index (the percentage of cells carrying a total chromosome set that differs from the one specified in the progenitor.)

**Example**

Below follows an example of a simulation with parameters corresponding to the colon cancer cell line DLD-1:

First, use the following values for the parameters at the start of the simulation:

| Number of generations | 1,5,10 |
| --- | --- |
| Non-disjunction probability | 5e-4 |
| Number of runs | 5 |
| Copy number | 2 |
| Use chromatid loss | ON |

For the selection values use the following fitness conditions:

| **Chromosome** | **Copy number** | **P(death)** |
| --- | --- | --- |
| ANY | 0 | 1.0 |
|  |  |  |

This condition removes all cells that have zero copies of any chromosome.

Then, run the simulation, wait for it to finish, when it has finished you should see a histogram over total chromosome number distribution as well as the graphs for the 5 different runs. You can now export the data by pressing ‘Save Generated Data’. Due to space limitations, no more than 10 generations will be drawn but simulations will proceed and data generated also for simulations of higher than 10 generation numbers.
